# Impacts of impaired face perception on social interactions and quality of life in age-related macular degeneration: A qualitative study and new community resources

**DOI:** 10.1371/journal.pone.0209218

**Published:** 2018-12-31

**Authors:** Jo Lane, Emilie M. F. Rohan, Faran Sabeti, Rohan W. Essex, Ted Maddess, Amy Dawel, Rachel A. Robbins, Nick Barnes, Xuming He, Elinor McKone

**Affiliations:** 1 Research School of Psychology, and ARC Centre of Excellence in Cognition and Its Disorders, The Australian National University, Canberra, Australia; 2 John Curtin School of Medical Research, The Australian National University, Canberra, Australia; 3 Discpline of Optometry and Vision Science, The University of Canberra, Bruce, Australia; 4 Academic Unit of Ophthalmology, The Australian National University, Canberra, Australia; 5 Research School of Psychology, The Australian National University, Canberra, Australia; 6 Research School of Engineering, The Australian National University, and Data61, Commonwealth Scientific and Industrial Research Organisation, Canberra, Australia; 7 School of Information Science and Technology, ShanghaiTech University, Shanghai, China; Bournemouth University, UNITED KINGDOM

## Abstract

**Aims:**

Previous studies and community information about everyday difficulties in age-related macular degeneration (AMD) have focussed on domains such as reading and driving. Here, we provide the first in-depth examination of how impaired face perception impacts social interactions and quality of life in AMD. We also develop a *Faces and Social Life in AMD* brochure and information sheet, plus accompanying conversation starter, aimed at AMD patients and those who interact with them (family, friends, nursing home staff).

**Method:**

Semi-structured face-to-face interviews were conducted with 21 AMD patients covering the full range from mild vision loss to legally blind. Thematic analysis was used to explore the range of patient experiences.

**Results:**

Patients reported faces appeared blurred and/or distorted. They described recurrent failures to recognise others' identity, facial expressions and emotional states, plus failures of alternative non-face strategies (e.g., hairstyle, voice). They reported failures to follow social nuances (e.g., to pick up that someone was joking), and feelings of missing out ('I can't join in'). Concern about offending others (e.g., by unintentionally ignoring them) was common, as were concerns of appearing fraudulent ('Other people don't understand'). Many reported social disengagement. Many reported specifically face-perception-related reductions in social life, confidence, and quality of life. All effects were observed even with only mild vision loss. Patients endorsed the value of our *Faces and Social Life in AMD* Information Sheet, developed from the interview results, and supported future technological assistance (digital image enhancement).

**Conclusion:**

Poor face perception in AMD is an important domain contributing to impaired social interactions and quality of life. This domain should be directly assessed in quantitative quality of life measures, and in resources designed to improve community understanding. The identity-related social difficulties mirror those in prosopagnosia, of cortical rather than retinal origin, implying findings may generalise to all low-vision disorders.

## Introduction

Age-related macular degeneration (AMD) is a progressive disease that causes central vision loss and reduced visual acuity [1]. AMD impairs many aspects of everyday functioning and independent life, such as ability to drive, read, and cook for oneself [2–3]. Previous research into reduced quality of life in AMD has focussed primarily on these areas of everyday function, without considering specifically the effects of poor face perception [4–9]. For example, the major quantitative questionnaire designed to assess macular-degeneration-related change in quality of life (the MacDQoL) [10] has no questions about face perception while including questions targeting multiple other domains (e.g., ability to engage in hobbies, self-care, or shopping); and its questions related to interactions with other people do not disentangle problems caused by face perception difficulties from problems caused by other aspects of AMD (e.g., inability to maintain a social life outside the home due to loss of driver's license). Similarly, the websites of national and international macular disease support organisations, to which patients may be referred by medical staff, provide information sheets and videos that focus on issues such as driving, reading and maintaining independence. These websites commonly show an image of a social scene with a central face blotted out by a black blob to illustrate the (supposed) effects of AMD on vision (e.g., https://ghr.nlm.nih.gov/art/large/age-related-macular-degeneration.png, accessed 28 March 2018), yet overlook the intricacies of potential difficulties with face perception and resulting problems with social interactions. These sites also do not address when in the course of macular disease progression a patient might begin to experience face-related social difficulties (e.g., in early stage AMD with mild vision loss, or only in late stage AMD with severe vision loss).

The implicit assumption in these previous approaches is that face perception problems in AMD are of relatively minor importance to patients' everyday lives. The present study was designed to provide the first evaluation of whether this assumption is true, via an in-depth exploration of the types of face-related experiences patients report in a qualitative interview.

There are several reasons to believe that, in fact, the functional importance of face perception problems in AMD might be high. First, it is well established that AMD impairs the ability to recognise the identity of faces and to see facial expressions, both in self-reports and formal laboratory testing [11–15]. Problems are particularly likely for faces seen small or in the distance, although can also occur even when the face is near (e.g., sized as during a natural conversation with an individual 1–2 metres away; e.g., [14,15]).

Second, there is strong evidence that poor face identity recognition is associated with negative psychosocial outcomes. Across the normal population range of young adults, poorer face identity recognition ability is correlated with increased social anxiety [16]. In prosopagnosia—a disorder in which face identity recognition is clinically impaired but at the brain rather than retinal level—social interactions, confidence, and quality of life can be severely affected. In a qualitative study of these effects, Yardley et al. [17] found all 25 participants described recurrent and at times traumatic social interaction difficulties, including: common failures to recognise family members, close friends, and work colleagues, which contributed to concerns about offending others, plus feelings of embarrassment, guilt and failure; particular social difficulties in groups due to not knowing who everyone was; resulting fear of and sometimes avoidance of social situations; dependence on others to help identify people; and long-term consequences that included a small social circle, damaged personal relationships (e.g., due to unintentionally ignoring a friend in the street), and reduced self-confidence. In low vision, there are no detailed studies of AMD patients, although the literature does contain a handful of quotes, from patients with a mix of eye diseases, suggesting similar face-identity-related social problems might occur (e.g., feeling embarrassed when not recognising others) [6,8].

Third, accurate face expression recognition is also important for normal social interactions. People use others’ facial expressions to judge how they are feeling (e.g., happy, angry), the intended meaning of their words (e.g., if they are serious or making a joke), whether they are engaged by the conversation or bored, and, ultimately, to decide how to respond [18]. Expressions also play a broader role in sending social signals (e.g., that a child genuinely needs help when displaying genuine sadness, or is merely pretending when displaying posed sadness), and misperceptions of such signals can lead to inappropriate social responses [19]. In low vision, again there are no previous studies that have examined expression-related social difficulties in any detail, in AMD or any vision disorders.

The present study explores the psychosocial impact of face perception difficulties in AMD, focussing primarily on problems arising from identity and expression recognition failures. We ask whether AMD patients might suffer the same identity-related difficulties in social interactions as seen in prosopagnosia. We also explore whether expression perception difficulties might result in additional problems, such as misinterpretations in social interactions. We also ask patients specifically about the *importance* of face perception to them, and explore whether *face* perception problems in particular—rather than all the other difficulties of living with AMD—impact their confidence, willingness to engage socially, and quality of life. Other topics we address in briefer form include: how faces appear to people with AMD (surprisingly, not a question that appears to have been previously investigated); whether patients attempt to use alternative non-face-based strategies for recognising people and emotions (e.g., voice, gait, body shape, hairstyle) and whether these are effective; problems with eye gaze and with facial cues to speech; and patient views on the potential value of technological help for improving face perception (e.g., smart glasses that could enhance face images to make them easier to recognise). Finally, we explore the questions of whether patients feel people around them understand their face-related vision difficulties, as relevant to the potential need for, and content of, community resources specifically focusing on face-related social difficulties.

Given the lack of any previous detailed information from AMD patients on how face perception affects their social interactions and quality of life, the appropriate methodology for a first investigation is qualitative, not quantitative. (Indeed, creating a *valid* quantitative measure to assess frequency and severity of problems cannot be done without first discovering the *types* of problems that patients experience [20]). We used interviews that were semi-structured and open ended. Questions were partly *a priori* (e.g., designed to examine similarity to previous findings concerning social effects of poor face identity recognition in prosopagnosia) but the study was also to a large extent exploratory. Thus, interviews included a mix of: questions asked directly of all patients; follow-up questions asked of some patients and not others depending on their previous responses; and spontaneous comments from patients.

Overall, our aim was to capture the *range* of experiences reported by AMD patients concerning the type and impact of their face recognition difficulties in everyday life. A key aspect of this was selecting patients to cover a wide range of vision loss—from very mild (e.g., still driving) to severe (legally blind)—to allow us to capture any phenomena that might be reported only by individuals at one end of this range. For example, perhaps it might be that only people with *moderate* or *severe* vision loss due to AMD report face perception problems that are bad enough to impact their social interactions and quality of life. Or, it might be that only people with *mild* vision loss report that others fail to understand their problems seeing faces.

We also included some standard quantitative questionnaires. These allowed us to more completely describe the sample (e.g., their depression and anxiety levels), and to allow replication of expected findings, including that self-reported everyday visual function should decrease with worsening visual acuity [13,21] and that AMD should be associated with a reduction in quality of life on the MacDQoL [22]).

In the second part of this article, we use the interview results to develop a community-targeted *Faces and Social Life in AMD Information Sheet*. A good understanding by others can potentially improve patients' quality of life by, for example, increasing empathy for the person living with AMD, allowing others to provide suitable practical help to assist social interactions, and decreasing the likelihood of others taking offence (e.g., if the person with AMD appears to ignore them or misunderstands their social cues). The information sheet is designed for AMD patients, family members, friends, and carers including, for example, nursing home staff. The wording style is aimed at the general public, that is, suitable for readers without medical or scientific expertise. It may also be of some value to medical professionals (e.g. ophthalmologists who wish to better understand the patient experience) or clinical psychologists and counsellors (e.g., if treating a person with AMD for depression or anxiety associated with social withdrawal). To accompany the information sheet, we provide a *Conversation Starter*, that guides family/friends/carers through a series of face perception questions they can ask the person living with AMD, to gain a better understanding of that *particular* person's day-to-day social experiences, and how the carer can best help them. Finally, we also provide a *1-page brochure*, suitable to be given to patients in vision clinics (e.g., by orthoptists), which include large-print information on a few key points and the web addresses at which the patient or family can find the Information Sheet and Conversation Starter. These new community materials are made available in S1 File and S2 and S3 Files.

## Method

### Participants

Participants were *N* = 21 AMD patients (all Caucasian; 16 female, 5 male; age *M* = 83.5 years, *SD* = 7.3, range = 66 to 92). To be eligible to participate, patients had to: (a) be diagnosed by a qualified ophthalmologist as having AMD in both eyes and no other eye diseases (to ensure any vision-related problems were attributable specifically to AMD; note non-visually significant lens opacity was permitted); and (b) not have dementia (patients who disclosed a diagnosis of dementia during recruitment were not invited to participate, and all tested participants demonstrated normal levels of cognitive functioning during interview). Additionally, (c) patients had to report, on initial contact, experiencing difficulties seeing faces in their everyday life: while all patients with moderate and severe vision loss would be expected to experience face perception problems [11–15], early-stage AMD patients might not and, it is necessary for patients to report face perception problems to then interview them about the effects of those problems on social interactions and quality of life (i.e., patients not yet experiencing face problems would add no data concerning our major aims).

Participants were recruited until (a) we had covered a wide range of severity of vision loss from mild to legally blind (Table 1), and (b) saturation was reached in the qualitative interview results (i.e., no new experience types were being reported, the standard criterion for sufficient sample size in qualitative research, e.g., [23,24]). Patients were recruited through advertisement or individual approach from author JL, via: The Canberra Hospital Eye Clinic; a private ophthalmologist’s clinical rooms; local radio interview discussing the study; or letter from the Macular Disease Foundation Australia to AMD patients living in the Canberra region.

**Table 1:** Individual patient details, with patients ordered by acuity (BCVA) in their best eye^1^.

Patient code & vision loss category	Age in years	Sex ^2^	Best Eye	Best Eye Visual Acuity		Best Eye Diagnosis
***Mild*** ***(<6/6 to 6/18)***				**BCVA**	**LCVA**	
P1	85	F	R	6/7.5	6/15	Dry AMD
P2	91 (93) ^3^	F	L	6/9.5	6/19	Wet AMD
P3	86	F	R	6/12	6/30	Dry AMD
P4	70	F	R	6/12	6/19	Wet AMD
P5	78 (78)	F	L	6/15	6/38	Wet AMD
P6	87	F	R	6/15	6/30	Wet AMD
P7	86	F	L	6/15	6/60	Dry AMD
P8	86	F	R	6/15	6/60	Early AMD
***Moderate*** ***(<6/18 to 6/60)***						
P9 ^4^	73	F	R	6/19	6/30	Wet AMD
P10	79	M	R	6/19	6/48	Dry AMD
P11	88	M	L	6/19	6/48	Wet AMD
P12	92	F	L	6/24	6/38	Early AMD
P13	66 (68)	F	L	6/24	6/60	Wet AMD
P14	82	M	R	6/38	6/48	Wet AMD
P15	84	F	L	6/38	6/60	Wet AMD
P16	78	M	L	6/60	6/95	Dry AMD
***Severe (<6/60)***						
P17 ^4^	89	F	R	<6/60 ^5^	^–^	Wet AMD
P18	82	F	R	6/75	6/150	Dry AMD
P19	92 (94)	F	L	6/75	6/120	Wet AMD
P20	90	M	L	6/75	6/190	Wet AMD
P21	91	F	L	6/190	<6/240 ^6^	End-stage AMD

Notes

^1^ Additional vision testing data, plus information for the other eye, in Table A S4 File.

^2^ Codes: M = male, F = female; L = left eye (i.e., OS, ocular sinister), R = right eye (i.e., OD, oculus dextrus); BCVA = Best Corrected Visual Acuity (high contrast letter stimuli), LCVA = Low Contrast Visual Acuity; "<" = worse than.

^3^ For the 4 participants with more than 6 months between interviews, age value in brackets gives the age at time of second interview. Table A S4 File provides acuity results on repeat test at time of second interview. None of the 4 participants' vision had degraded sufficiently to change them into a more severe vision loss category.

^4^ Participants P9 and P17 did not do the second interview due to ill health.

^5^ P17 did not have a vision assessment at the ANU and her visual acuity (BCVA only) was reported by her ophthalmologist. For correlations (Table 2) her BCVA value was entered as 6/60 or logMAR +1.0.

^6^ LCVA listed as <6/240 indicates the patient could not read all letters on the largest line of the LCVA chart.

Concerning demographics, the sample was generally middle-class and financially secure. For the 20 patients willing to answer financial questions, none disagreed with the statements “I have enough to pay my household bills” and “I have enough to pay for household repairs or help needed in the house”; only 4 disagreed with “I can afford to buy what I want”; 6 agreed with “I cannot afford to do things I would enjoy”. Regarding highest education level, 7 had a university qualification, 7 another tertiary qualification (e.g., certificate or apprenticeship), 5 secondary school and 2 primary school. Eighteen patients resided in their own house (8 still with a spouse), and 3 in assisted accommodation (e.g., nursing home). All reported regular contact and support from others (e.g., spouse, adult children, grandchildren, carers). Three participants were still driving.

Most patients were tested across three sessions, lasting up to 2 hours each. They were not paid, beyond reimbursement of travel to the university. The research was conducted in accordance with the Declaration of Helsinki. Ethics approval was obtained from the Human Research Ethics Committees of Australian Capital Territory (ACT) Health (protocol ETH.10.13.291) and Australian National University (protocol 2013/386). Participants' written consent was obtained, following explanation of the study and possible consequences; this included specific consent for the qualitative interviews to be audio recorded, and for publication of de-identified quotes.

### Acuity, and criteria for mild, moderate and severe vision loss categories

Best Corrected Visual Acuity (BCVA) was measured by a qualified orthoptist using a retro-illuminated LogMAR chart mounted on a stand conforming to the ETDRS standard format [25]. Vision loss categories were defined using BCVA cut-off values from the International Statistical Classification of Diseases and Related Health Problems 10^th^ Revision [26]. *Mild* vision loss refers to BCVA poorer than 6/6 (normal vision), down to 6/18. *Moderate* refers to BCVA poorer than 6/18, down to 6/60. *Severe* refers to BCVA poorer than 6/60. To give concrete interpretation to the acuity values, in Australia, a standard driving licence requires BCVA better than 6/12, and 6/60 is legal blindness.

Patients were ranked (Table 1) and grouped based on *best*-eye visual acuity. This was on the grounds that AMD can affect the two eyes to different extents (in our sample, correlation between acuity in the two eyes was only *r* = .28), and it is functional acuity in the best eye which is likely to be the primary determiner of how well the patient can see faces in everyday life. This is because the brain preferentially attends to input from the eye providing the higher-quality input and tends to ignore input from an eye providing lower-resolution input; see evidence from amblyopia [27], or after laser surgery where the two eyes are given different corrections for close and far viewing [28]. S4 File provides: detailed information about both eyes (including BCVA, low contrast visual acuity LCVA, AMD type and stage); details of full vision assessments; and evidence that best-eye BCVA was indeed the most appropriate measure on which to rank patients' everyday vision ability.

### Quantitative questionnaires: Everyday visual function, and psychological wellbeing

Overall level of everyday visual function was assessed using the National Eye Institute Visual Functioning Questionnaire (NEI-VFQ-25; Interviewer Administered Format plus appendix [21]). State (i.e., current) level of depression and anxiety were assessed using scales validated for elderly participants: the Geriatric Depression Scale-15; GDS-15 [29], and the Geriatric Anxiety Inventory; GAI [30]. AMD-related change in quality of life was assessed using the Macular Degeneration Quality of Life Questionnaire; MacDQoL [10], this instrument uses patients' ratings across multiple domains (e.g., ability to engage in hobbies, household tasks, travel outside the house, shopping, perform self-care) of how their life would change if they did not have AMD (but everything else in their life remained the same) multiplied by their rating of the importance of that domain to them. All questionnaires were administered verbally.

### Qualitative interviews

Interviews were one-on-one. They were conducted at the ANU, the patient's place of residence, or (in a few cases) on the telephone. Each patient took part in one, or both, of two interviews, each lasting 30–45 minutes.

To explore the range of patient experiences, Interview 1 (all 21 patients) was semi-structured and open ended. The initial questions asked directly of all patients are listed in S5 File. S6 File gives examples of participants’ very different responses to a given initial question, and the corresponding variation in follow-up questions. Topics addressed were those described in the Introduction.

Interview 2 (19 patients, see Table 1) was primarily concerned with obtaining patient feedback on material for potential inclusion in our *Faces and Social Life in AMD Information Sheet*. We drafted a list of possible facts and statements, based on findings after testing most patients on Interview 1. We then asked patients in Interview 2 whether they did or did not endorse each fact/statement as useful to include in the Information Sheet, and to provide comments as needed (e.g., where they thought clarification or qualification was needed). In some cases, Interview 2 also revealed additional patient experiences, and/or included additional follow-up and clarification questions arising from their Interview 1 responses. Time delay between Interviews 1 and 2 ranged from same-day testing up to 2 years; vision assessment was repeated for the 4 patients with longer than 6 months delay (with none having moved to a more severe vision loss category across the delay; see Table A S4 File).

### Interview data coding

Interviews were transcribed verbatim, combined across Interviews 1 and 2, and entered into NVivo software (QSR International Pty Ltd, Version 10, 2015) to assist with data collation. Patient experiences from the transcripts were coded into themes [31], using a mix of a bottom-up (inductive) and top-down (theoretical) approaches. For bottom-up analysis, three authors independently read 6 interviews (3 from Interview 1 and 3 from Interview 2) from patients spread across the three AMD severity levels, and extracted emergent themes relevant to the present research questions (i.e., content related to face perception and/or its effect on social interactions and quality of life); coding strategies between authors were reviewed, and themes chosen were based on consensus negotiation [32]. Themes were also redefined (e.g., two sub-themes combined), and additional themes were developed, in a top-down manner, to ensure adequate coverage of all the specific topics we wished to address (e.g., emotion perception; technology preferences), and to allow comparison to previous findings in the literature (e.g., whether AMD patients experience the same types of identity-related social-interaction problems reported in prosopagnosia). JL then coded the interview transcripts from each patient into the final themes, including whether the patient had experienced that type of phenomenon or not, together with the piece/s of quoted text relevant to that experience. EM cross-checked the coding, with discrepancies resolved via negotiation. In addition to the initial coding of full interview transcripts to themes, multiple text search queries were conducted for each theme to avoid missing any data.

## Results

### Quantitative measures of visual function and psychological wellbeing

Table 2 presents sample-descriptive results for quantitative scales, including across our full patient sample, and subgroup means for patients with mild, moderate and severe vision loss (ICD-10 criteria [26] as used in Table 1). Table 2 also shows correlations with acuity (best-eye BCVA from Table 1, converted to logMAR; note higher logMAR scores indicate poorer vision).

**Table 2:** Patient results and comparison values for quantitative questionnaires.

Measure	Scale comparison values	All patients *N* = 21 *M*(*SD*)[range]	Correlation with acuity (best-eye BCVA) (*r*) ^7^	Means for vision loss subgroups ^8^		
				Mild *n* = 8	Mod *n* = 8	Severe *n* = 5
**Everyday Visual Function** NEI-VFQ-25 (full scale) ^1^	100 = no difficulty 0 = maximum difficulty	46.9(12.1) [22.2–69.8]	–.47*	50.0	48.4	39.5
Item A6 (face identity) ^2^	100 = no difficulty 0 = maximum difficulty	32.1(26.4) [0–100]	–.58**	43.8	34.4	10.0
Item Q11 (expression) ^3^	100 = no difficulty 0 = maximum difficulty	55.6(32.7) [0–100]	–.37	66.7	59.4	31.3
**Depression** (GDS-15) ^4^	0–4 = normal 5–9 = mild depression 10–15 = moderate to severe depression	4.5(2.7) [1–10]	+.12	4.8	3.9	5.2
**Anxiety** (GAI) ^5^	0 = minimum anxiety level ≥ 11 indicates Generalised Anxiety Disorder	3.5(4.4) [0–14]	+.45*	2.3	2.6	6.8
**Quality of Life (**MacDQoL) AMD-related change ^6^	+3 = improved QoL 0 = no impact of AMD –9 = maximum reduction	-3.9(1.7) [-0.8-(-6.6)]	–.41^†^	-3.7	-3.1	-5.4

Notes

^1^ Composite score on NEI-VFQ-25 (National Eye Institute 25-Item Visual Functioning Questionnaire Interviewer Administered Format plus Appendix) is the average of the vision-targeted subscale scores, excluding the general health rating question [21].

^2^ NEI-VFQ-25 Item A6 = ‘Because of your eyesight, how much difficulty do you have recognizing people you know from across a room?’

^3^ NEI-VFQ-25 Item Q11 = ‘Because of your eyesight, how much difficulty do you have seeing how people react to things you say?’ For this question *N* = 18 as three patients did not answer (P6 and P18 said they did not know and P7 said it depends on distance).

^4^ Cut-offs from [34], GDS [29].

^5^ Cut-off to identify Generalised Anxiety Disorder in older adults from [30].

^6^ MacDQoL [10] measures macular-degeneration-associated *change* in quality of life (QoL), assessed across 23 domains. Scores are weighted impact score, calculated by multiplying patients' rating for AMD-change (–3 = maximum reduction in ability in that domain to +1 = improvement) by their rating of importance of that domain to them (0 to 3), and averaging across the 23 items (or fewer if a domain did not apply to patient, e.g., work).

^7^ Correlation directions (with acuity expressed as logMAR) are such that worsening visual acuity is associated with worse everyday visual function (negative r), increasing anxiety and depression (positive r), and poorer quality of life (negative r). For comparison of correlation to zero (two-tailed)

** = p < .01

* = p < .05

^†^ = p < .07

^8^ Mild, moderate and severe vision loss groups, defined by best eye high-contrast visual acuity (BCVA, Table 1) using ICD-10 criteria [26] (see Method).

As expected, Table 2 demonstrates impairment in self-reported everyday visual function. All individual patients reported meaningful everyday impairment on the NEI-FVQ (highest score = 69.8 where 100 is no impairment). Self-reported NEI-VFQ function correlated significantly with objective vision level, with function worsening with worsening acuity (significant negative correlation with BCVA). We also found impairments specifically on the two NEI-VFQ items that are relevant to face perception (Question A6 ‘because of your eyesight, how much difficulty do you have recognizing people you know from across a room?’; and Question 11 ‘because of your eyesight, how much difficulty do you have seeing how people react to things you say?’). Mean scores for both items were well below the no impairment level, and every patient indicated impairment on the identity and/or the expression question (i.e., the highest scores of 100 indicated in the range data in Table 2 came from different patients). Both face-relevant items showed correlations with objective acuity that were in the predicted direction, significantly so in the case of the face-identity-related item (Question A6).

For psychological wellbeing measures, Table 2 shows worsening acuity correlated significantly with increasing anxiety. Depression did not correlate linearly with worsening acuity (a finding consistent with evidence of psychological adjustment to chronic disease [33]). Results for the MacDQoL showed a sizeable AMD-associated reduction in quality of life on average (i.e., mean score of –3.9 where 0 is no impact), with a close-to-significant correlation between worsening acuity and greater AMD-associated reduction. Note there are no face-related-item data provided for the MacDQoL in Table 2 because the measure includes no items from the face domain.

### Qualitative experience of AMD patients

To illustrate the range of experiences patients reported in the interviews, we use a mix of quotes (from which irrelevant information has been removed, and any names changed) and tables containing the percentage of patients reporting certain experiences. These percentages are minimum values in many cases (marked with a '+' in Tables 3–7); this is because, in a semi-structured interview procedure, not all patients are necessarily asked directly about all experience types, meaning additional participants within our sample may have endorsed the experience if explicitly asked about it. Our reason for reporting concrete numbers at all—which is unusual in a qualitative interview study—is to provide information on whether a given difficulty was reported, say, only by patients with severe vision loss, or also reported by patients with mild vision loss.

**Table 3:** Difficulties seeing faces in AMD, how faces appear to patients, and problems with face identity and expression recognition.

	% of Patients Reporting this Experience ('+' indicates minimum value, i.e., not all patients asked directly about the experience)			
Description of Experience	Mild ^1^ (*n* = 8)	Moderate (*n* = 8)	Severe (*n* = 5)	Total (*N* = 21)
**Difficult to see faces**				
AMD has made it harder to see faces	100	100	100	100
Unable to see faces properly even at close (conversational) distances (e.g., 1–2 metres)	38+	50+	60+	48+
Faces are hard to see on TV	50	88	80	71
**How faces appear**				
Faces appear abnormal in some way:	50+	100+	80+	76+
- Faces appear blurred	50+	88+	80+	71+
- Faces appear distorted/have missing parts	13+	38+	40+	29+
- Other experiences (e.g. central black blob; black flecks)	25+	25+	60+	33+
**Variability in seeing faces**	88+	88+	80+	86+
Impacted by lighting	88+	88+	60+	81+
Other factors (e.g., varies with time of day)	50+	50+	60+	52+
**Specific problems with facial identity & expression**				
Problems recognising facial expressions	100	100	100	100
Problems recognising face identity	100	100	100	100
- failing to recognise people you know (false negatives)	88+	88+	100	91+
- ‘recognising’ people you don’t know (false positives)	50+	88+	80+	71+

Notes

^1^ Mild, moderate and severe vision loss groups, defined by best eye high-contrast visual acuity (BCVA, Table 1) using ICD-10 criteria [26] (see Method).

**Table 4:** Alternative strategies that AMD patients try to use, and their effectiveness.

	% of Patients Reporting this Strategy ('+' indicates minimum value, i.e., not all patients asked directly about the strategy)			
Description of Strategy	Mild (*n* = 8)	Moderate (*n* = 8)	Severe (*n* = 5)	Total (*N* = 21)
**Identity recognition (visual strategies):**				
Body shape/size	38+	75+	80+	62+
Walk/gait	38+	63+	20+	43+
Hair (colour, length, hairstyle)	13+	50+	60+	38+
Clothing	25+	38+	20+	29+
**Identity recognition (nonvisual strategies):**				
Voice	100	75+	100	91+
Context (expecting certain people in certain locations)	50+	25+	100	52+
**Expression/emotion recognition:**				
Body language	25+	38+	20+	29+
Auditory cues to emotion (e.g., tone of voice, hearing laughter or crying)	100	63+	100	86+
**Affecting both identity and expression:**				
Proximity (e.g., wait for person to come closer so patient can see their face more clearly)	63+	100	40+	71+
**Effectiveness of these strategies:**				
My strategies don't always work	25+	62+	80+	52+
**Reliance on other people**				
Others help (e.g., tell me who is approaching)	75+	88+	100	86+

**Table 5:** Difficulties with, and changes to, social interactions arising from poor face perception.

	% of Patients Reporting or Endorsing this Experience ('+' indicates minimum value, i.e., not all patients asked directly about the experience)			
Description of Experience	Mild (*n* = 8)	Mod (*n* = 8)	Severe (*n* = 5)	Total (*N* = 21)
**Identity domain**				
- "Some people with AMD may appear disengaged, this may be because they cannot see who is in a room." (*N* = 15)	100	100	100	100
- When I don't recognise others, I worry they will think I’m rude or unfriendly	50+	63+	60+	57+
- To avoid false recognition of someone I don't actually know, I am cautious / hesitant / noncommittal (e.g., I don't say people's names, wait for them to speak first)	38+	25+	80+	43+
- To try to avoid giving offence to people I know by ignoring them, I'm indiscriminately friendly (e.g., I smile at everyone)	25+	25+	0+	19+
**Expression domain**				
- "People with AMD may be unable to see a person’s facial expressions i.e., whether someone looks happy, sad or bored. Because they cannot see facial expressions, they might miss social cues. For example, someone might be looking bored but the person with AMD can’t see this so they keep on talking, or a person might be just having a joke and is smiling when they say something, but the person with AMD takes it seriously." (*N* = 16)	86	100	100	94
- Patient gave specific example/s of above from their own experience	38+	50+	20+	38+
**Face perception domains in combination**				
- Social interactions are slowed or take more mental effort	63+	50+	80+	62+
- Particular difficulty in groups	38+	25+	40+	33+
**Responses to mistakes**				
- I apologise	63+	38+	60+	52+
- I explain I have vision loss (or wear a vision impaired badge)	88+	88+	100	90+
- I use humour (laugh it off)	25+	63+	60+	48+
- I sometimes let it go/pretend there is no problem	75+	88+	100	86+
- I sometimes feel bad (embarrassed, frustrated, sad, upset)	100	100	100	100
- I worry what other people think of me and how they judge me	63+	88+	60+	71+
- Others usually respond positively to my mistakes (e.g., humour, kindness, helpful)	50+	50+	60+	52+
- Others can get angry/upset when I make mistakes	38+	25+	20+	29+
- I sometimes can't tell how others respond (because I can't see their expressions)	50+	63+	40+	52+

*Notes*: For the two statements listed in quotes, a subset of patients (N = 15, and N = 16) were read these statements, as part of the pre-testing phase for the patient information sheet (within Interview 2), and asked whether they agreed with them.

**Table 6:** Other face problems: Eye gaze and facial speech.

	% of Patients Reporting this Experience ('+' indicates minimum value, i.e., not all patients asked directly about the experience)			
Description of Experience	Mild (*n* = 8)	Moderate (*n* = 8)	Severe (*n* = 5)	Total (*N* = 21)
**Problems with eye gaze** e.g., can't make eye contact; can't see where other people are looking; can't see eyes	50+	38+	80+	52+
**Problems with facial speech** e.g., AMD has made it harder to follow face-to-face conversations *but not* phone conversations (ruling out a hearing loss origin); can't lip read anymore (for patients with previous lip-reading skill); can't see mouth	38+	63+	40+	48+

**Table 7:** Impact of reduced face perception on social life, confidence, and quality of life; plus lack of understanding by other people.

	% of patients reporting experience or responding ‘yes’			
Description of Experience	Mild (*n* = 8)	Moderate (*n* = 8)	Severe (*n* = 5)	Total (*N* = 21)
**Social life: missing out and withdrawal**				
- Missing out (e.g., can't join in with the jokes; didn't realise my old friend was at the funeral)	63+	75+	100	76+
- I disengage/isolate/withdraw in social situations	63+	38+	100	62+
- I am less willing to have social interactions due to my reduced face perception	63	25	60	48
**Face-specific effects on reduced confidence, quality of life**				
- Problems seeing faces has reduced my confidence	75	38	80	62
- Problems seeing faces has reduced my quality of life	75	63	100	76
**Other people don't understand**				
- Other people don't understand how AMD impacts my vision	75+	50+	80+	76+
- I worry other people think I'm faking it	50+	25+	40+	38+

### Difficulties seeing faces, facial appearance, and variability in face perception

Table 3 collates reports of difficulties seeing faces. Results show that all patients, regardless of their residual visual acuity, reported their vision loss had made it harder to see faces. Problems were described as particularly acute at longer distances (e.g., across a room; also see the NEI-VFQ results in Table 2), but nearly half of patients reported having problems seeing faces clearly even at conversational distances (1–2 metres), including three mild patients.

Concerning how faces appear visually to people with AMD, Table 3 shows three-quarters of patients spontaneously mentioned one or more ways in which faces no longer looked normal. The most common aspect mentioned was that faces appeared *blurred* (or equivalent terms such as ‘*unclear’*, or ‘*low-definition’*). This blur meant that patients could not always see internal features clearly. For example, one patient described the interviewer, sitting less than 2 metres away, as having ‘*two holes for the eyes*’ (P1; mild). Additionally, nearly a third of patients mentioned seeing shape distortions and missing parts in the face. The nature of these varied: one patient said ‘*The distortion is quite bad … on one side the mouth goes up and the eyes keep disappearing … or looks blurred and moving a bit’* (P2; mild); another said the ‘*features are kind of deformed*, *jumbled* … *it’s as if the face were on a piece of sheeting or something and somebody grabbed it from behind and pulled it like that [simulating a sheet being grabbed] and it just went all scrunched up’* (P9; moderate); another said *‘I can see the right hand side of you … not the left’* (P19; severe). One third of patients also reported other general visual disturbances (e.g., black flecks, lights and floaters) that would impact on the appearance of faces. Three patients mentioned seeing a black blob in the centre of their vision (a common illustration of the supposed perceptual effects of AMD; see Discussion), while one patient (P18; severe) specifically said they did *not* experience a black blob in the centre, instead describing their experience as like ‘*looking through a screen’* or ‘*looking through black tulle*’.

Table 3 also shows that many patients mentioned variability and inconsistency in how well they could see faces. Lighting was reported as a relevant factor by most (e.g., one example was that it was harder to recognise faces with the light behind them). Eleven patients said they prefer strong lighting, with faces harder to see in lower light levels. Three said the opposite, namely that they are light sensitive and prefer low lighting. Two said their light preference varies, i.e., sometimes they require strong light and other times they are light sensitive. One of these latter patients commented *‘This is one of my husband’s big bug bears*, *because he just can’t understand why one minute I want light and the next minute I don’t’* (P13; moderate). Some patients identified other factors associated with variability in how well they can see faces, including time of day (e.g., improvement as the day goes on), and treatment phase (i.e., pre/post injection if being treated with ranibizumab for Wet AMD). One patient said: ‘*Sometimes I can see*, *sometimes I can’t’* (P19; severe).

### Difficulties with face identity and expression recognition

Table 3 shows that all patients reported their difficulties seeing faces resulted in problems recognising both face identity (who other people are) and facial expression. Importantly, the problems were not limited to those with moderate and severe vision loss, but also occurred in mild vision loss.

For facial identity, both false negatives and positives were common. Almost all patients had experienced problems recognising people they know (false negatives). This included reports of failing to recognise good friends and close family members. Problems occurred even with mild vision loss, for example ‘*I have had it happen*, *it’s very embarrassing* … *the other day I didn’t even recognise my son* … *within a yard or two of me and I didn’t recognise him*, *he said “Mum*, *it’s David*!*”‘* (P2; mild). In general, patients with more severe vision loss reported such failures occurring more frequently. When asked ‘Do you find that you fail to recognise people you know?’, responses included ‘*Oh all the time* (P18; severe)*’*, and ‘*I would pass people by in the street that I know* *very* *well*. *It’s very embarrassing* … *they’ve said to me “hey Mary how are you*?*”* … *I would not have a clue* … *I don’t mean to be rude but I just can’t see them’* (P21; severe).

Many patients also reported falsely ‘recognising’ people they did not actually know (false positives), such as approaching a person to say hello thinking they were a friend, only to find they were a stranger. When asked ‘Have you said hello to someone thinking it was someone you knew, and it actually wasn’t?’ example responses included ‘*Yes and it’s not them at all*. *It’s someone totally different*, *yes*, *that becomes a bit embarrassing’* (P19; severe), and ‘*Yes [laughs] … Someone I knew very well*, *I went up and started having a conversation with them and they looked at me blankly*. *And you know when I was closer*: *“yeah*, *you’re not who I thought you were”*. *I apologised to them*, *but what they thought*, *I don’t know’* (P14; moderate). These experiences of false positives and negatives in everyday life closely match quotes describing identity-related failures in prosopagnosia [17,35].

Turning to facial expressions, comments suggested expression perception was even more severely affected by AMD, earlier in the progression of the disease, than identity recognition. When asked: *Has AMD impacted your ability to see a person’s facial expressions*? example responses were: ‘*Yes*, *I think that was one of the first things that went*, *not actually see the expression’* (P5; mild), and ‘*As far as expressions go*, *that’s something that’s gone’* (P16; moderate), and ‘*Well you don’t get facial expressions with this disease’* (P18; severe).

### Alternative non-face-based strategies, and their effectiveness

Problems identifying faces and recognising facial expressions would not have serious implications for social interactions if AMD patients were able to use other strategies to successfully recognise people and their emotions. However, this was commonly not the case.

Table 4 lists various alternative strategies that patients reported trying to use (not necessarily successfully). For identity recognition, the most common visual strategy mentioned was using body shape/size followed by walk/gait, hair/hairstyle and clothing. Patients also reported two nonvisual strategies, voice recognition and context. Context was identified as both a help ‘*When it’s a normal meeting it’s not so bad because most of them sit on the same tables’* (P20; severe) and a hindrance (when people are seen out of their usual context or when the patient was expecting someone else). Most patients reported using multiple strategies simultaneously, for example ‘*I look at the way people are walking … mainly their gait and their general appearance … maybe for the women I look at their hair … their hairstyles … their size and behaviour … and then of course if they speak it’s voice recognition’* (P18; severe). These identity-related strategies were identical to those that prosopagnosics report trying to use [17,35,36]. For expression, the two main strategies reported for trying to understand other people's feelings and emotions were using body language (e.g., a sad or angry posture) and voice: ‘*The tone of voice gives them away*. *Mostly it’s reflected in people’s voices whether they are in a happy mood or a grumpy mood’* (P11; moderate). A strategy used by many patients, relevant to both identity and expression, was proximity, i.e., moving closer to others, or waiting for others to approach to try to improve the clarity of the face. Overall, these alternative strategies involved either looking at visual information that survives low resolution vision relatively well (e.g., because the body is larger than the face, or because determining hair colour and length requires only coarse spatial information), or using nonvisual information (auditory cues, context).

Importantly, patients reported that the non-face-based strategies they tried were often ineffective. Table 4 shows half the patients reported their suite of strategies failed: for example, ‘*I can make some terrible mistakes … [my strategies] help*, *they are certainly not fool-proof’* (P11; moderate), and ‘*I’m not sure that [my strategies] are very effective at all’* (P1; mild). Moreover, even when patients initially described their strategies as ‘effective’, further interview responses revealed patients generally meant the strategies worked ‘most of the time’ or ‘in some contexts’ (e.g., for close family members). Patients also reported factors that can impair the effectiveness of their strategies; these factors included crowds, how often they see the person (i.e., level of familiarity), and the fact that some strategies are unreliable due to changes in the environment. Environmental changes included a participant who misidentified her own husband because he had recently lost weight and his body shape had changed (P13; moderate), and another who said ‘*One of the ladies at church had long curly hair and she got it all chopped off and I didn’t have a clue who she was until she spoke’* (P14; moderate). Finally, the effectiveness of alternative strategies appears to decrease (Table 4) as AMD severity level increased. This may be because visual cues that survive mild loss of visual acuity well (e.g., body shape) become too blurred to be useful in moderate-to-severe AMD.

The failure of AMD patients' alternative strategies meant they often reported being reliant on other people for assistance. In total, 86% of patients reported others helped sometimes, by naming the person aloud (e.g., ‘here comes Bill’ or ‘James is sitting at the back of the room with his wife’) or describing emotions (e.g., ‘Mary looks sad today’ or ‘Jan was smiling when she said that Mum’). Additionally, however, 7 patients reported that they would appreciate more help of this kind.

### Difficulties with, and changes to, social interactions

Table 5 collates patient responses concerning the ways in which their face perception problems alter their immediate social interactions.

#### Identity recognition

In the identity domain, the social-interaction impacts reported by AMD patients were strikingly similar to those reported in prosopagnosia [17,35,36].

First, patients experienced difficulties and disengagement in social situations, due to not knowing who was present. P16 (moderate) said: *‘I walk around the block and past the club*. *A lot of the times I walk in and see who’s in there*. *If anyone speaks to me I stop*, *have a yarn to them for a while*. *But if nobody speaks to me I don’t stay*, *I just walk out again’*. P17 (severe) said: *‘I walk into the room of a morning and they’re all sitting there waiting to do yoga and I think “why can’t I see them*?*”* … *someone might call out “Oh*, *hi Jenny” well*, *I don’t know where they* are’. P12 (moderate) noted the need to rely on others to achieve social engagement: *‘the younger ones* … *I was really pleased because they came looking for me to speak to me*, *whereas I wouldn’t have found them’*. Concerning disengagement due to not knowing who was in the room (Table 5), P4 (mild) said *‘that sort of puts it in a nutshell actually’*.

Second, many patients were concerned about embarrassing themselves and/or offending others, and often changed their behaviour in attempts to avoid negative social interactions. To avoid the embarrassment of false-positive recognitions (i.e., saying hello to someone they did not know), many patients mentioned becoming more cautious, hesitant or noncommittal, and avoiding using names. P17 (severe) said ‘*I wait until I am spoken to*’. P9 (moderate) said poor face recognition has made her ‘*a bit more careful … a bit more tentative*’. P18 (severe) said ‘*I try now to discipline myself not to identify*, *not to say “oh this is my friend Jan”… I say non-committal things like “Hello how are you*?*”*, *not “I don’t believe we’ve met”‘*.

Concerning false negatives (i.e., failing to recognise familiar people), patients were very concerned about the impact on others. Most worried about appearing rude, unfriendly, or standoffish. P14 (moderate) said *‘I know I walk past people and ignore them because I don’t recognise them … I am sure that I upset people* … *What they think of me I don’t know*, *it worries me’*. P8 (mild) said others probably think she’s ‘*snobby*’. P15 (moderate) said she feels *‘embarrassed [about] cutting them dead* [i.e., appearing to deliberately ignore them] *or whatever they think*’. P5 (mild) said *‘If you go out and you meet someone and have a conversation with them*, *and then the next time you meet them you don’t even recognise them*, *I imagine it would be unpleasant for the person* … *you were getting on famously and then next time you wouldn’t recognise them*. *I would think I would hurt people’s feelings’*. Two participants described situations where they directly knew they had offended another person. In the most extreme case, P18 (severe) reported *‘I go to craft on Sundays and this lady came in*. *She would usually come and talk to us*, *and then go over there and read the paper … Anyhow*, *on this occasion she didn’t come over and I didn’t know she was there* … *I went over and got a glass of water and when I walked past her to come back she yelled*, *“**You don’t even speak to me*!*”*. *She frightened the life out of me*, *I didn’t even know she was there*, *and I said to her*, *I am so sorry I didn’t even see you there because I’m vision impaired*, *you know that*. *And anyhow*, *there was a bit of a discussion* … *I was crying and I said I didn’t mean to ignore you’*.

As a way of dealing with concerns about failing to recognise familiar people, a few patients took the strategy of being indiscriminately friendly (e.g., smile at everyone) to avoid potentially offending anyone. For example, P8 (mild) said ‘*I just smile at people because I think*, *well [laughs]*, *I* *might* *know them’* and P13 (moderate) said ‘*I just smile at everybody*. *There are probably people down the street who think “I wonder who that mad woman is who is smiling at me*?*” … [but] it is just easier … then you don’t offend anybody*, *and if you smile at someone and they* *do* *know you and they want to stop and speak to you then they will’*. This contrasted with the tendency of most participants to be more cautious in their dealings with other people (to avoid false positives).

This pattern is similar to that reported in prosopagnosia, where Yardley et al. [17] also found that in most participants the tendency is to become more cautious towards other people, while in a smaller subset the tendency is the opposite. Also note that patients' emotional responses to making mistakes in general varied: while all reported feeling bad about mistakes in some way (Table 5), not all patients reported they specifically felt embarrassed (replicating results in [15]). Example quotes included: ‘*I don’t know about embarrassment*, *but it can be frustrating’* (P20; severe); ‘*I haven’t felt the embarrassment one but the frustration is definitely there’* (P4; mild); *‘No*, *I don’t find embarrassment’* (P19; severe); and *‘Well*, *no I would never feel embarrassed’* (P18; severe).

#### Expression perception

Other types of social difficulties reported by AMD patients can be related to problems specifically in expression perception. This includes failures to correctly understand others’ emotions, failures to understand what specific event had elicited an emotion, failures to pick up on whether others were joking or serious, inability to tell when others wished to speak to them or had got bored with their conversation and it was time to change topic, and/or worrying about whether they might be making these types of mistakes. Fifteen patients endorsed a statement describing that these types of problems can occur in AMD (Table 5), and eight gave examples. P9 (moderate) said *‘It can be a bit embarrassing if you don’t pick up correctly [that someone is sad]*, *and just be happy and jolly*, *and that might not be appropriate at all’*. P5 (mild) said *‘With one doctor*, *I said to my daughter when we came out “Boy*, *he was a bit cranky wasn’t he*, *did I do something to upset him*?*” she said “No*, *he was just making a few jokes to try and break the ice”*. *But I thought*, *to me he sounded as if he was cranky and I couldn’t work it out*. *But my daughter said “No he was smiling”‘*.

One patient (P13; moderate) emphasised the normal social cues that had been lost with AMD: *‘[normally] if you’ve wounded someone’s feelings you can actually see*, *“oh I’ve hurt her” or “I shouldn’t’ve said that” or “I shouldn’t have said it the* *way* *I said it”*. … *[Or] you can actually see that they are enjoying the conversation*. . . . *whereas if you can’t see their face*, *you don’t have a clue’*. Similarly, another said *‘I would never speak to anybody first now whereas I used to always*, *because I find if you speak to someone most times they’ll speak back to you*, *but I haven’t yet to learn to tell by their voice whether they're* *pleased* *that you are speaking to them or not so I don’t do it anymore’* (P5; mild).

Inability to see rapid dynamic changes in expression also resulted in failures to understand what specific event had elicited an emotion. For example, P16 (moderate) said *‘If I am talking to people and someone there is laughing and carrying on*, *I know they are as happy as buggery* [i.e., very happy], *but I can’t see their face to see what*, *you know to see* *when* *their face changed’*.

#### Multiple domains: Slowing, difficulty in groups, eye gaze and facial speech

Other social difficulties reported by patients can arise from a combination of face perception problems across the domains of identity, expression, eye gaze and/or facial speech.

Thirteen patients reported social interactions had become slower or required more mental effort. This could arise from many specific factors, for example: taking longer to realise who people are; the increased cognitive load of needing to remember who is sitting where in a group; having to 'work out' what caused someone to laugh rather than perceiving this directly. P11 (moderate) said his impaired face perception meant ‘*I don’t interact quickly*, *I am now much slower in making decisions when talking to them’*. P13 (moderate) said that during conversations *‘Sometimes when someone says something to you*, *you have to click your brain in to register what they are saying … I’m concentrating so hard on their face that sometimes words just go away*’.

Additionally, a third of participants raised the issue that social interactions can be particularly difficult in crowds or groups. For example, P20 (severe) said he found conversations hard to follow in groups, and P4 (mild) said *‘[Social situations] are very difficult particularly in a crowded room if you are at a function’*. Theoretically, this finding is consistent with the fact that group situations pose the most challenging setting for face perception. That is, to fully engage in a group social interaction, one needs to be able to: rapidly identify all members of the group; pick up immediately on rapid changes of expression or emotion and what events these were in response to; use eye gaze cues to pick up on social signals such as when it might be your turn to speak or when the group's attention has shifted elsewhere [37]; and potentially use facial speech cues to help understand what others are saying (particularly in a noisy environment [38]).

Specifically concerning eye gaze and facial speech, Table 6 shows approximately half our patients mentioned problems relevant to these domains. For eye gaze, quotes suggested problems were particularly prevalent in group situations. For example, P16 (moderate) said ‘*If I’m sitting around talking to anyone in a circle or anything*, *I can’t see their eyes’* and P20 (severe) said ‘*Looking at someone at the other side of the table … I can’t see if they are looking at me [as opposed to someone else at the table]’*. For using facial motion to help understand speech, P17 (severe) said ‘*I can’t see the mouth* *at all*, *no way’*, and P2 (mild) who had been trained in lip reading following partial hearing loss said her face-to-face conversations are ‘*tied up with my lip reading*, *so very difficult’*.

#### Responses to mistakes

Social interactions were also altered by the need to respond to mistakes. Where patients made explicit mistakes that were obvious to the person affected by the error (e.g., failing to recognise a familiar person, saying hello to a stranger, or failing to realise someone is upset), patients employed a variety of strategies for social repair. As shown in Table 5, they routinely apologise. Depending on the circumstances, patients sometimes explain they have vision loss—*‘I’m sorry my eyesight’s bad’* (P15; moderate), or *‘I have macular degeneration*, *I am having a bit of a problem with recognising faces’* (P10; moderate)—although also note two of our patients chose not to disclose their vision loss beyond close family. Patients sometimes use humour to laugh off their mistakes: *‘[with] some of the people I know really well*, *I can joke about it with them’* (P14; moderate). Patients sometimes attribute their mistake to another source (e.g., pretending they had a memory failure), particularly with people they don't know well, or where the patient does not disclose or does not wish to spend the time on a detailed conversation explaining AMD. On other occasions, patients report trying to ignore their mistakes and just get on with it without making a social repair attempt: ‘*I just try to look as though I know what I am doing’* (P8; mild), and *‘There is nothing you can do about it*, *you just go with it’* (P12; moderate).

Patients also reported experiencing a variety of responses to their mistakes from others. Overall, patients reported that others were commonly helpful and kind. For example, *‘Nobody takes offence*, *they just give a little chuckle’* (P21; severe), *‘[Others] do the best that they can to help me’* (P12; moderate), and *‘Most people are very considerate and tolerant’* (P14; moderate). However, more than a quarter mentioned having experienced occasions on which others got angry and upset. Importantly, half also said there were occasions where they had no idea how the other person felt (e.g., because they could not see their expression); this is relevant to validity of one item on the MacDQoL [10] (see Discussion).

Despite using often-successful social repair mechanisms, AMD patients reported that a number of negative emotions remained associated with making mistakes. As Table 5 shows, many mentioned feelings of embarrassment, frustration or sadness. Many also reported worrying what others think of them and how they judge them (e.g., being perceived as rude, or stupid).

#### Severity of vision loss

An important observation (Table 5, plus example quotes above) is that the face-related social difficulties, and changed social behaviour, in AMD were not limited to those with severe vision loss, but were also experienced by patients even with only mild vision loss.

### Longer-term impact on patients’ social life, confidence, and quality of life

Table 7 summarises information about the longer-term effects of poor face perception on patients' social life, confidence, and quality of life. Negative impacts were common.

#### Missing out

Concerning the quality of social life, three-quarters of our patients reported examples of missing out on the full quality of social experiences available to people with normal vision. Three said that due to their poor face perception they ‘*can’t join in*’ in social interactions (P15, P16, P19; moderate and severe), and even P1 with very mild vision loss (best-eye BCVA acuity of 6/7.5) said ‘*you’re not with the rest of the crowd*’ and *‘You’re not getting out of a conversation perhaps what you would normally get out’*. In examples that patients found particularly upsetting: P18 (severe) said *‘I sat there [at a social function] for fully two hours not knowing who the people at the table were*, *and that was pretty distressing’*; and P12 (moderate) said *‘At things like funerals where you know a lot of people and you don’t recognise them … it’s disappointing afterwards when you hear that someone was there that you would have liked to have seen’*.

Five participants mentioned missing out when watching TV or movies, for example when watching a drama programme they lose track of who’s who and so cannot follow the story properly (P12; moderate), or because in a panel show *‘Sometimes I find it hard to follow the interchange’* (P10; moderate).

#### Social withdrawal, passivity

Many patients reported that the social difficulties, and/or the decreased enjoyment to be obtained from social settings, had led to an increased tendency to withdraw or isolate themselves, and to become more socially passive. This could occur within an individual social situation: for example, P19 (severe) said *‘[when you make a mistake recognising others] you want to go back and put yourself in a corner or in your room somewhere away’*. It also resulted in half of patients agreeing that poor face perception had contributed to them being less willing overall to have social interactions than before they had AMD (Table 7). For example, P20 (severe) said *‘I'm less interactive*’. P5 (mild) said *‘I'm more passive*, *definitely less interactive and tend to stay in the background*. . . . *I don’t socialise anymore [with new people]’*. P4 (mild) said ‘*If I am going somewhere where there is going to be lots of people I sometimes don’t want to go*’. P16 (moderate) said *‘You become a sort of a loner’*. And P18 (severe) said *‘I'm more mousey now … I go up [to the social area] and sit down quietly*, *whereas one time I would have been the president [laughs] … I think it’s made me more introverted’*.

#### Face-specific effects on confidence and quality of life

Recall that, while it is well established that AMD lowers confidence and quality of life ([5,39]; also Table 2 in present sample), our novel question here is whether patients experience reductions *that they see as specifically associated with their poor face perception*, as opposed to the many other difficulties they experience in AMD. Table 7 shows that, when asked directly whether problems seeing faces had reduced their confidence, more than half of patients agreed. When asked directly whether problems seeing faces had reduced their quality of life, more than three quarters of patients agreed.

Effects on quality of life included, in some cases, strong feelings of loss. For example, P17 (severe) said ‘*It’s* *very* *important [to be able to see other people’s faces] I want to see them and it’s very distressing that I can’t … I want to see them’*. And P8 (mild) who had previously worked assisting a politician said *‘I always prided myself*, *it was one of my best things when I was working for a politician*, *is that I could recognise all the people who came in to talk*, *I would say “this is so and so”* … *It used to be my pride*, *I could recognise people and give him the name … [now I can't do that anymore]* *it feels as though it’s not me**’*.

### Resilience

In addition to the difficulties described above, it should be noted that most patients revealed examples of resilience, some degree of coming to terms with their face problems, and/or determination to fight against social isolation. Quotes include: *‘[not being able to see facial expressions] used to make me feel upset* … *it was as if I’d lost something*, *lost a person sort of*, *but I’ve kind of got used to it now* … *You either get up and go or you sit in your chair and die and I think I’d rather get up and go’* (P5; mild); ‘*I think it [social withdrawal] could happen to people but I don’t let it happen to me* … *I imagine that AMD could [make me more passive] and maybe it will get me that way eventually*, *at the moment I am still fighting it’* (P2; mild); ‘*I’ve got on with life the best I can*’ (P14; moderate); ‘*Unless you sort of make an effort [socially]*, *you could have a very miserable life*, *and I’m just not prepared to have a very miserable life*’ (P13; moderate).

### 'Other people don't understand'

Table 7 shows that most patients felt others did not understand how AMD affects their vision, and how hard it was for them to see faces clearly. In some cases, this occurred even when the other person was well aware of the AMD: *‘They know what I am like*, *at that minute they just forget’* (P16; moderate), and *‘They don’t realise [my vision’s] deteriorated yet’* (P8; mild). In some cases, it reflected the other person having difficulty understanding that AMD can affect fine vision as needed for face recognition, but without externally-visible damage to the eyes, and without impairing coarse vision as needed to walk around. For example: *‘A couple of people have specifically said to me*, *in appearance*, *you don’t look as though you are having problems with your vision*, *your eyes look perfectly normal’* (P5; mild), and *‘I have lots of friends and family even who say to me “well how can you see that and you can’t see something else*?*”‘* (P13; moderate).

This lack of understanding resulted in more than a third of patients raising concern that others think they are 'faking it'. For example: *‘I think she [my carer] thought I was just putting it on a bit you know’* (P8; mild), and *‘He [my son] had been a bit doubtful I think about it*, *but that [my failure to recognise him] has quite convinced him now that faces are distorted for me’* (P2; mild). This problem was not limited to patients with only mild vision loss, where others might perhaps be expected to be least appreciative of AMD difficulties (e.g., because the patient can still navigate well around the environment). Examples from moderate vision loss included: *‘sometimes you sort of think they’re doubting that you even have a problem’* (P13; moderate); and *‘I feel like a fraud [when I need to ask for help]’* (P9; moderate). Even patients who were legally blind worried that others didn't believe they had a real problem seeing faces: *‘I do think people think you are faking it a bit’* (P18; severe).

### Relative importance to patients of faces versus other aspects of vision loss, and of facial identity versus expression

Three questions addressed how important patients perceived their face problems to be, including relative to the many other visual domains affected by AMD (e.g., ability to read, drive, or cook). At the very beginning of Interview 1, we asked an open-ended question—‘Which areas or tasks have been made harder because of your AMD?’—to record how many patients would *spontaneously* mention face perception difficulties as amongst their most important everyday problems: 38% did so (3 mild, 4 moderate, 1 severe). This compared to 81% mentioning reading and 62% hobbies (e.g., knitting, sewing, crosswords and writing) as the two most common topics raised, and to rather smaller percentages for some domains included in the MacDQoL [10] (e.g., only 5% for enjoying meals and 14% for shopping; see S7 File for details). Later we asked *directly* for ratings of importance. In the identity section of Interview 1, in response to ‘How important is seeing other people's faces to you?’ 15 patients indicated High/Very High, 5 said Medium, and only 1 said Low. In the expression section, in response to ‘How important is it for you to be able to see another person's facial expressions?’, 16 patients indicated High/Very High, 4 said Medium, and only 1 said Low (a different participant from the person who chose Low for identity). Together, results of these three questions indicate that face perception is of high importance to most AMD patients, and also of no lesser importance than several domains currently included in the MacDQoL [10].

We also asked patients which was more important to them, recognising facial identity or recognising facial expressions: 67% said it is more important to be able to recognise who a person is (i.e., identity); 24% reported the two are equally important; and 10% said it is more important to see a person’s facial expressions.

### Patients’ views on possible technological help

Our results so far indicate that technology that could improve patients' face perception and recognition would have the potential to improve their social interactions and quality of life. Our qualitative interviews thus addressed patients' willingness to try various types of future technological assistance. Most patients (71%) said they would be willing to use computer facial recognition software that could say aloud the name of other people in the environment. While a commercial product offering this service is available (for 100 learned faces [40]), note the named-by-computer approach has some disadvantages compared to improving a participant's own perception: naming aloud may interfere with hearing an approaching person speak, and is also only useful only for identity recognition (a running verbal commentary on dynamic aspects of faces like expressions or eye gaze would not be suitable for patients). We thus also asked about *image enhancement*, which has the potential to improve perception of all aspects of faces. We explained that, in image enhancement, patients would view faces on computers, tablets, or smart glasses [41,42] with the faces digitally altered in ways that have been reported to improve low-resolution face perception (e.g., making the face larger [15,43]; increasing the contrast of medium and high spatial frequencies [44,45]; or caricaturing the face, i.e., exaggerating its appearance away from the average to make identification easier [46–48]). Participants were on the whole very positive about trying image enhancement technology if and when it became available, particularly on TV and/or computer screens (90% support; e.g., one patient mentioned this would be useful when skyping his family), and via smart glasses (90% support). Only one patient (P16) was not interested in any type of electronic device, saying technology ‘*left me behind*’. Several patients noted that hand-held screens (e.g., phones, tablet computers) would not be valuable because they needed their hands available for balance, carrying things, and in some patients for using a mobility walker; thus, a hands-free option such as smart glasses was preferred. Several participants highlighted potential concerns regarding the weight of smart glasses on the face, and the likely expense of the technology (even in our largely middle-class sample).

### Development of information sheet, conversation starter and brochure

In Interview 2, all but one participant asked indicated that an information sheet concerning the effects of AMD on face perception and social interactions would be useful. Responses from Interview 1 were used to draft potential points for inclusion in the information sheet (see Method). The final version contained concepts that were directly endorsed by patients for inclusion (for details, see S8 File), plus some additional points widely raised by patients (Tables 3–7) which we had not included in the draft but emerged as important themes once we had the full data set to analyse. Feedback on a final draft was also obtained from the Macular Disease Foundation Australia (via their Research Officer and Medical Affairs Manager), concerning appropriate formatting and language for macular degeneration patients.

Our final community resource materials are in S1–S3 Files. The content of the *Faces and Social Life in AMD Information Sheet* highlights the core issues arising from the patient interviews: how faces might look to people with AMD; how early in disease progression problems could potentially arise; the types of social problems patients might experience that result from difficulty seeing faces; that *individual* experiences can be highly variable; and that the patient's experiences are genuine and normal for AMD. The sheet also aims to help others around the patient (e.g., family members, friends, carers, nursing home staff) appreciate compassionately why a patient may sometimes appear to behave in a socially odd manner (including appearing rude or unfriendly); to understand why they may have become more passive or disengaged in social settings; and to provide suggestions for how others might be able to help in concrete terms (e.g., by naming people as they approach). To give greatest accessibility to vision impaired patients (as advised by Macular Disease Foundation Australia), the information sheet has been prepared in large font, all plain text (no italics), and maximum contrast (plain black text on white background).

Our accompanying *Conversation Starter* has questions for others to ask the individual person living with AMD. These are aimed at enabling AMD patients to describe in detail to family, friends and carers (e.g., nursing home staff) their own *individual* experiences in seeing faces, the impact this is having on their social interactions and social life, and what they would like other people to do to help. Finally, we provide a tri-fold *1-page brochure* that can be given to patients at vision clinics, or picked up by family members, containing directions to finding the information sheet and conversation-starter online; the brochure and information sheet are provided in both A4 and US-letter paper sizes.

## Discussion

This study found that poor face perception in AMD impacts patients' psychosocial functioning in multiple ways. Concerning failures of identity recognition, patients with AMD revealed the same social interaction difficulties previously reported in prosopagnosia [17,35,36]. These included: failures to recognise even highly familiar people (e.g. family members); false recognitions of unfamiliar people; common feelings of embarrassment or frustration about these errors; concerns about offending others; and resulting changes in social behaviour, with most patients becoming more cautious around others to avoid making false positive identifications, and a few taking the opposite approach of being friendly to everyone to avoid false negatives. In addition to these identity-related problems, AMD patients experienced further difficulties associated with their problems perceiving facial expression and eye gaze. This included not being able to tell when others were joking, failures to be able to read others' emotional states, and inability to tell when others were making eye contact. There was also some evidence suggesting problems with facial speech. Taken together, the problems perceiving multiple aspects of face information resulted in patients commonly feeling they could not fully join in, or were not part of the group. Many reported social withdrawal or reduced enthusiasm for social events. Confidence and quality of life were reduced and, crucially, patients attributed at least some of this reduction specifically to face perception problems. On explicit ratings of importance, most patients rated face perception as high. Finally, psychosocial problems were seen even in patients with only mild vision loss, and patients commonly reported that others did not understand their face perception problems, and that they sometimes worried about being seen as a fraud.

Overall, these results argue that the importance of face perception difficulties to everyday life in AMD is higher than has been implicitly assumed in previous approaches, including quality of life research and in community information websites.

We now discuss a number of specific outcomes of our study in more detail, including open questions and limitations of the study where relevant.

### Implications for the current Quality of Life instrument for macular degeneration (MacDQoL)

A key implication of our findings is that problems with face perception in AMD contribute to social difficulties and reduced quality of life, over and above all of the other functional visual problems that occur in AMD (i.e., inability to drive, engage in hobbies, remain independent, etc.). This argues the MacDQoL [10] would benefit from adding a specific item about face recognition.

The MacDQoL is the only quantitative questionnaire designed specifically to measure change in Quality of Life due to macular degeneration. It is widely used [49], is available in 14 languages (https://www.healthpsychologyresearch.com/information/currently-available-translated-questionnaires/MacDQoL-macular-disease-dependent-quality), has good reliability, and has been validated overall (e.g., scores correlate with level of vision loss; [22,50]). However, its 23 items include no questions addressing the domain of face perception. Additionally, while it does include questions relevant to social relationships with others ('closest personal relationships'; 'family life'; 'friendships and social life'), these questions do not mention faces, and patient responses could equally be related to other non-face difficulties: for example, a patient's personal relationship with their spouse may be negatively impacted by the fact they can no longer contribute to household tasks; or a patient's friendships and social life may be reduced due to loss of driver's licence. Wording for a face-domain question for the MacDQoL could be along the general lines of asking the patient how much better they could see other people's faces (e.g., recognising who they are, or what they are feeling) if they did not have macular degeneration, and then following up with the usual MacDQoL question structure by asking participants how important seeing other people's faces is to them. (Unfortunately, we cannot suggest here precise wording that would match the MacDQoL format, because the questionnaire and its precise format is copyright).

Our present study also revealed a problem with one of the existing items on the MacDQoL [10]. Q15 asks patients about how much better other people would react to them if they did not have macular degeneration (with choice between 5 "amount" options ranging from very much better to worse). However, consistent with evidence that AMD patients often cannot *see* how others react to them, due to poor facial expression perception, 7 of our 21 patients said they could not answer this question—indeed, one said "*How do I know*?". The inability to see how others react is a well-established aspect of vision loss of multiple types (e.g., as reflected in the NEI-VFQ’s inclusion of the item 'Because of your eyesight, how much difficulty do you have seeing how people react to things you say?'), further arguing that asking patients about how much macular degeneration has affected how others react is not a useful wording.

In sum, our present results argue the MacDQoL could be improved in validity by (a) adding a question about quality of life in the domain of seeing faces, and (b) removing or rewording Q15.

### Improving community and patient information about AMD

Concerning community information about AMD, our interview results have revealed two key findings: poor face perception in AMD is qualitatively related to many difficulties in social interaction; and patients commonly feel that others don't understand these difficulties. This argues that one way to improve patients' quality of life is to improve community understanding of face-related social difficulties in AMD.

Our *Faces and Social Life in AMD Information Sheet* (and accompanying conversation stater and brochure) provides a much higher level of detail concerning face perception and social interaction difficulties than previously-available public material. For example, beyond noting that faces can be hard to see, macular disease organisations including the Macular Society (https://www.macularsociety.org/), the American Macular Degeneration Foundation (https://www.macular.org/), and the Macular Disease Foundation Australia (https://www.mdfoundation.com.au) have previously given no information about how this can affect social interactions. Vision Australia, which deals with all vision disorders including total blindness (http://www.visionaustralia.org), has an information page briefly explaining that vision loss can result in difficulties with social interactions due to poor face perception. This page mentions problems seeing facial expressions and maintaining eye contact, but does not mention facial identity. As the present results show, problems with identity recognition can have very important negative impacts on social interactions and social life (also see [17,35] in prosopagnosia). Additionally, the Vision Australia page does not refer to macular degeneration specifically, nor explain that AMD patients might sometimes experience face-related social interaction problems even when their vision is still otherwise quite good (e.g., good enough to drive).

Our hope is that our new community materials will help others around the patient (e.g., family members, friends, carers, nursing home staff) appreciate compassionately why a person living with AMD may sometimes appear to behave in a socially odd or changed manner (including appearing rude, unfriendly, or unusually passive), and to help to assist with maintaining social engagement in practical ways (e.g., by naming people as they approach).

### Qualitative results provide a basis for future development of a quantitative questionnaire

An important limitation of the present study is that we have not aimed to examine the *frequency* or *severity* with which different types of face perception and social interaction difficulties occur. There are many research questions of scientific interest that can be addressed only with access to such quantitative information. For example, these include: (a) in multiple regression, *how much* of the decreased quality of life in AMD is uniquely related to face perception and social interaction problems over and above, say, the contributions from other aspects of vision loss (e.g., loss of ability to read or drive) or general age-related difficulties (e.g., other health problems, death of spouse or old friends; (b) whether a potential intervention (e.g., psychological treatments for social anxiety; advice to disclose AMD-related face problems to others; technology to improve face perception) produces a significant improvement in social interaction and quality of life by comparing pre- versus post-intervention scores; (c) whether certain face perception or social interaction difficulties might be more severe in certain ophthalmological states (e.g., wet versus dry AMD) or perceptual states (e.g., the patient experiences blur-plus-distortions in the face, or only blur); and (d) the extent to which AMD patients have insight into their precise level of deficit (i.e., by correlating quantitative self-report of everyday face problems with lab-based measures of face ability), noting that insight is limited in normal vision [51,52] but perhaps may be better in patients given the potential benefit of internal comparison to their earlier abilities before AMD.

Development of a validated quantitative questionnaire to measure face-related social interaction difficulties is beyond the scope of the present study. Importantly, however, the qualitative analysis presented provides a crucial first step towards this end, by providing evidence on the *types* of face perception and psychosocial difficulties experienced by AMD patients. This detailed understanding of the types of experiences that can occur is an essential step towards developing a valid quantitative measure. Research that creates quantitative self-report measures without prior qualitative understanding can result in validity problems. For example, in the 8-item self-report questionnaire [15] developed to supposedly assess AMD patients' insight into their level of face recognition difficulties, four items do not actually ask simply about perceived disability in face perception/recognition *per se*: two ask about *emotional responses to mistakes*; and two ask about *alternative strategies* and wrongly assume that the only alternative strategy is *voice*.

Our qualitative results indicate that important domains to assess quantitatively, depending on the specific interests of the researcher, could include: frequency with which everyday face recognition problems are experienced (including identity, expression, eye gaze, and facial speech); degree of success or failure of alternative strategies to recognise people and their emotions other than by their face; frequency and severity of various types of social interaction difficulties related to impaired face perception; severity of negative emotional response to making errors (noting our participants varied on this, with some highly embarrassed and trying to avoid errors at all costs, and others more inclined to shrug off many of their mistakes); and severity of face-related impacts on social functioning, confidence, and quality of life. Potentially, items for a formal questionnaire could be based on the experience categories in Tables 3–7 and/or include rating-response versions of questions included in our Conversation Starter (S1 File).

To fully develop a quantitative self-report questionnaire from the present results would require large-sample testing to determine psychometric properties for any proposed instrument, including reliability (e.g., Cronbach's alpha, test-retest), factor structure, and convergent and divergent validity. For example, there may be separate factors for severity of face perception problems and severity of negative emotional responses to those problems; if so, we would expect *face perception* to correlate most strongly with acuity and lab-based face tests, while *emotional response* might correlate less with acuity and more with personality attributes.

### How widespread might social-interaction problems be in patients with only mild vision loss?

A potentially surprising finding from the present study was the consistent reports of face-related social interaction difficulties even in AMD patients with mild vision loss. While it may of course be the case that these occur *quantitatively* less frequently than in patients with more severe vision loss (an issue which requires development of a quantitative questionnaire to evaluate), *qualitatively* the problems were the same as in the moderate and severe vision loss categories. In terms of face identity failures, perhaps the most striking was our second mildest patient, who despite having visual acuity of 6/9.5 (best-eye BCVA), reported recently failing to recognise her own son standing next to her. Concerning expressions, a patient with acuity 6/15 said expressions were ‘*one of the first things that went*’. In terms of social interaction effects of poor face perception, even our best-acuity patient (acuity 6/7.5) said ‘*you’re not with the rest of the crowd … you’re not getting out of a conversation perhaps what you would normally get out*’.

Noting that we specifically recruited AMD patients who reported on initial phone contact that they experienced face recognition problems in everyday life, an open question is how widely spread face-related social interaction difficulties will be amongst mild vision loss patients. Predictions vary depending on the possible cause.

First, predicting that social problems in mild-vision-loss AMD patients would be common, is a potential role for *low-contrast visual acuity*. Low- and high-contrast acuity are highly correlated (r = .93 in our sample) but absolute acuity performance is always poorer for low contrast than high contrast stimuli (see lower LCVA than BCVA for patients in Table 1; also for normal-vision observers [53,54]. Acuity for static, high contrast letter stimuli is not fully reflective of performance in real-world viewing conditions [55,56]. Faces, in particular, are dynamic stimuli seen in changing conditions of lighting and contrast, and also intrinsically contain much low contrast information, such as the shape of the boundary between the nose and the cheeks for face identity, or the presence of frown lines in the forehead for expression. Thus, the BCVA measure, and the ICD-10 [26] categories based on it, is likely to underestimate the absolute degree of functional vision loss relevant to perceiving faces. The greater absolute vision loss implied by LCVA suggests social interaction difficulties would likely be widespread in other 'mild' patients.

Alternatively, predicting that social problems in mild-vision-loss AMD patients may be more restricted is the idea that there may be something special about mild patients who experience face perception problems often enough to noticeably impact their social interactions. One hypothesis is that there is something specific about the pattern of retinal damage in our patients that account for face perception difficulties severe enough to cause social interaction problems (e.g., perhaps such patients tend to have their relatively-well-preserved acuity supported by a single small region of preserved retina within the fovea, rather than by peripheral vision); note that, while we have detailed retinal data for our patients (S4 File), we cannot yet evaluate this idea empirically because we did not recruit mild-vision-loss patients *without* any face problems for the present study. Another hypothesis is that there might be an effect of *the other eye* for our patients. While other-eye acuity did not predict functional vision levels across our full sample (e.g., on the NEI-VFQ; see S4 File), it is of some note that of our 7 mild vision loss patients, 6 had severe vision loss in the other eye (i.e., BCVA worse than 6/60; see Table A S4 File). Thus, it cannot be ruled out, for example, that while typically patients preferentially use input from their best eye and ignore input from the poorer eye [27,28], our particular mild patients might be more likely than average to experience breakthroughs into attention from the poorer eye, and that these impair ability to perceive faces (e.g., if the severely-impaired other eye sends input suggesting unexpected movement in the face, such as a distortion or a part disappearing or reappearing, noting that movement attracts attention).

### Are negative consequences of poor face identity recognition due to having a disease not known to the general public?

In addition to the major issues arising from our study discussed above, a number of brief points arise concerning a variety of topics.

Yardley et al. [17] hypothesised that, in prosopagnosia, low public awareness of the disorder at the time may have been a major contributor to feelings of embarrassment, guilt, and failure. Awareness of AMD in Australia is very high (in 2011, 80% of people aged over 15, and 92% of people aged 50+ years were aware of macular degeneration, and 73% understood it is a disease of the eyes [57]). Despite this, we still found feelings of embarrassment, guilt, and concerns about being perceived as a fraud (also see [9]). It thus seems that the critical variable here is not public awareness of the medical condition *per se*. Instead, it may be that AMD is an *invisible condition* (noting experiences of being treated as fraudulent or exaggerating are reported in other medical conditions not easily visible to others, e.g., endometriosis [58,59]), and/or that others do not have sufficient information to understand the *specific* ways that AMD actually affects vision (i.e., that it impairs fine vision tasks far more than coarse-vision tasks). This argues improving the patient experience requires increasing understanding of the *detailed* symptoms of AMD, rather than merely its existence.

### How faces appear to patients with AMD: Blurred, distorted, variable, and often not the central black blob of traditional illustrations

Surprisingly, no previous study seems to have asked AMD patients in any detail what faces look like to them. Taylor et al. [60] recently provided the first detailed self-reports about visual appearance in (dry) AMD, covering visual experience in general rather than specifically faces. Across 29 patients with geographic atrophy, the most highly reported descriptors were *blur* (45%), *missing parts* of the image (34%) and *distortions* (24%). For faces, our present results agree in broad terms, with *blur* the most common phenomenon reported (71%) and reports of *distortions and/or missing parts of the face* the next most common (29%; patients with dry and wet AMD). Also in agreement with Taylor et al. [60], we found considerable heterogeneity in reported appearance across different patients.

These results are important because they indicate that the most common illustration of how the world is supposed to appear to patients with AMD is inappropriate (e.g., National Eye Institute, NEI, https://nei.nih.gov/health/examples; also [61]). The NEI illustration would often be viewed by newly-diagnosed patients wondering what to expect as their AMD progresses, and shows a black or grey blob completely hiding faces located at central vision. However, in [60], only 2 of 21 patients said this type of image reflected a good depiction of their vision. Here, only 3 of our 21 patients spontaneously mentioned seeing a blob in their central vision, and one patient explicitly said she did not, and overall it was striking how many more patients reported blur as a key feature of their visual experience than a central blob. More broadly, patients felt that others didn't understand how faces looked to them, and also how variable this was (e.g., with lighting). Our *Faces and Social Life in AMD Information Sheet* provides a more accurate description of the range, and variability, of facial experience that patients might experience. This may be useful for medical staff explaining AMD to newly-diagnosed patients (e.g., orthoptists), for patients themselves, and also for family, friends and nurses to better understand why faces are so difficult for patients with AMD.

Finally, the reports of variability have important implications for the design of lab-based tests to assess objective face processing ability. Patients report that how well they see faces can vary substantially across lighting conditions, and across time (e.g., time of day, pre- vs. post- treatment with ranibizumab). This argues that, to obtain an accurate objective score for lab-based face ability, it may be important to test the patient on several different occasions, at different times of day, and with different lighting conditions on the faces.

### Alternative strategies and importance of faces to successful real-world person recognition

The present study is the first in low vision to investigate the success or otherwise of non-face-based strategies for recognising others and their emotions. The key findings were that AMD patients report that, in everyday life, they attempt to use a wide range of alternative strategies (hairstyle, body shape, gait, voice, context) but these non-face-based strategies commonly fail. Specifically, we found that body language and tone of voice are not sufficiently strong cues to emotional state to fully enable normal social interactions. We also found that cues such as body shape, gait, and hairstyle are not sufficiently strong or reliable cues to identity to enable accurate recognition of who people are. This latter result is of some interest given occasional claims by vision science researchers that hairstyle is sufficient to support identity recognition (e.g., based on findings such as [62]). Our present results, in contrast, confirm previous findings from prosopagnosia (e.g., [17,35,36,63]), and also from low-resolution images (security camera video of walking people with faces covered [64]), that the ability to accurately perceive *face* information is crucial to reliable person recognition in everyday life, with other cues offering only partially useful information.

### Generalisation to other low-vision conditions

Finally, our results are relevant to low-vision conditions beyond macular degeneration. As with AMD, studies discussing effects of other types of vision loss on social interactions, social life, and quality of life have not disentangled, in any detail, effects specifically related to face perception problems (e.g., [8,65,66]). The details of how faces appear to patients with different disorders will vary (e.g., see description of patient visual experience in glaucoma [67]). However, all vision disorders producing low visual acuity will result in problems seeing faces clearly. There is no reason to think that the types of difficulties in *social interactions* we have reported here are in any way related to the specific type of retinal damage that occurs in macular degeneration. Indeed, the (identity-related) social interaction difficulties in AMD closely mirror those present in prosopagnosia, where face recognition problems do not originate in the eye at all, but rather in the brain. Thus, it is highly likely that any eye disorder resulting in low vision will produce qualitatively similar social difficulties to those revealed in AMD, together with the same concomitant effects of missing out socially, tendency to social withdrawal, reduced confidence, and reduced quality of life specifically associated with face perception difficulties.

## Supporting information

S1 File New community resources (faces and Social Life in AMD information sheet in A4 and US-letter versions and conversation starter). (PDF)

S2 File New community resources (faces and Social Life in AMD brochure), A4. This brochure is formatted to print and fold in tri-fold, size A4. Note: The guardian of the child whose image is in the brochure has given written informed consent to have their child’s image published under a CC-BY license. The child whose image is in the brochure was not a participant in the study.
(PDF)

S3 File New community resources (faces and Social Life in AMD brochure), US-letter. This brochure is formatted to print and fold in tri-fold, size US-letter. Note: The guardian of the child whose image is in the brochure has given written informed consent to have their child’s image published under a CC-BY license. The child whose image is in the brochure was not a participant in the study.
(PDF)

S4 File Full vision assessment information. This includes the rationale for ranking patients' functional vision based on best-eye BCVA and Supplementary Table A and Table B.
(PDF)

S5 File Interview 1 initial questions. (PDF)

S6 File Interview 1 example of different follow-up questions to initial questions, arising from different patient responses. (PDF)

S7 File Relative importance of face perception domain compared to domains currently included in MacDQoL. Includes Supplementary Table A and B.
(PDF)

S8 File Interview 2 results for patient endorsement of content included in faces and Social Life in AMD information sheet. Includes Supplementary Table A.
(PDF)
