# Supplementary material for: Impacts of impaired face perception on social interactions and quality of life in age-related macular degeneration: A qualitative study and new community resources
Source: PLoS One. 2018 Dec 31;13(12):e0209218. doi: 10.1371/journal.pone.0209218 (PMC6312296; doi:10.1371/journal.pone.0209218)
Supplement: S1 File — (PDF) [file pone.0209218.s001.pdf]

Supporting/Supplementary Information S1 File

Impacts of impaired face perception on social interactions and quality of life in age-related macular degeneration: A qualitative study and new community resources

Jo Lane[1], Emilie M. F. Rohan[2], Faran Sabeti[2,7], Rohan W. Essex[3], Ted Maddess[2], Amy Dawel[1], Rachel A. Robbins[4], Nick Barnes[5], Xuming He[6], Elinor McKone[1*]

Revision Submitted to PLoS ONE for PONE-D-18-19813*, 7 December 2018*

Supporting/Supplementary Information

Supplement 1: New community resources (Faces and Social Life in AMD Information Sheet in A4 and US-letter versions and Conversation Starter).

Supplement 2: Full vision assessment information, including rationale for ranking.

patients' functional vision based on best-eye BCVA (Includes Table 1 and Table 2).

Supplement 3: Interview 1 initial questions.

Supplement 4: Interview 1 example of different follow-up questions to initial questions, arising from different patient responses.

Supplement 5: Relative importance of face perception domain compared to domains currently included in MacDQoL (Includes Table 3 and 4).

Supplement 6: Interview 2 results for patient endorsement of content included in Faces and Social Life in AMD Information Sheet (Includes Table 5).

Supplement 7: References for **s**upplementary materials 2-7.

Supplement 1

New community resources

Included on pp 3-11 are our new:

- Faces and Social Life in Age-related Macular Degeneration Information Sheet (pages 3-5 are in A4 format and pages 6-8 are in US letter format)
- Faces and Social Life in Age-related Macular Degeneration: A Conversation Starter

The brochures ready to be printed in tri-fold form are available as separate supplementary files S2 (A4 size) and S3 (US-letter size).

Faces and Social Life in Age-related Macular Degeneration

An information sheet for people living with AMD,
and their family, friends and carers

AMD can make it hard to see faces which may lead to some common mistakes:

Everyone is different, but depending on lighting and distance, faces can look blurred or distorted. Because faces can be hard to see:

<u>You might not recognise people you know:</u>

- The other day I didn’t even recognise my son … he was within a yard or two of me and I didn’t recognise him.
- I pass by people in the street that I know <u>very</u> well.

<u>You might accidentally say hello to someone you don’t know:</u>

- I went up to someone I knew very well and started having a conversation with them. They looked at me blankly, and I realised they were not who I thought they were. It was very embarrassing!
- I am more careful now and wait for others to speak first.

<u>You might use other information to help but it doesn't always work:</u>

- I look at the way people are walking, their size, their hairstyles … and then of course if they speak it’s voice recognition.
- I can make some terrible mistakes, the other information helps but it is certainly not fool-proof.

<u>You might not see facial expressions:</u>

- You can't see if you’ve wounded someone’s feelings, so you don't realise ‘oh I’ve hurt her, I shouldn’t have said that’.
- I thought my doctor was upset with me and I couldn’t work it out, but my daughter said he was having a joke just to crack the ice.

<u>You might find crowds hard:</u>

- I find social situations very difficult particularly in a crowded room
- I find crowds uninteresting … if I can’t see and can’t place people then it’s a bit of a waste for me.

Sometimes, these problems can start when your vision loss is quite mild.

People don’t understand how AMD impacts my vision:

- It’s difficult, they forget I have AMD.
- They don’t realise my vision has deteriorated.
- I worry people think I am faking. They say ‘your eyes look perfectly normal’.
- They wonder why I can’t recognise people but I can walk around ok

Other people might think I'm rude or unfriendly:

Many people with AMD worry that others think they are ignoring them on purpose.

- People are mostly kind about mistakes (they help or laugh it off), but sometimes they do take offence: When I walked past Jenny and didn't recognise her, she yelled ‘you don’t even speak to me!’

Face problems can make social situations difficult:

Some people with AMD may <u>appear passive or disengaged</u> because they cannot see who is in a room.

- My old friend Tony was at the funeral and I would have loved to talk to him, but sadly I had no idea he was there.
- I sat there [at a social function] for fully two hours not knowing who the people at the table were, and that was pretty distressing.
- I find social situations can be a bit tricky when you can’t see what other people are feeling.
- You can’t feel completely part of what’s going on.

Some people <u>withdraw or lose confidence</u>:

- I don’t socialise anymore … I would think I would hurt people’s feelings … they thought you were getting on famously and then next time you don't recognise them … it’s easier not to put yourself in that situation.
- I am more mousey now ... I go up to the social area and sit down quietly, whereas one time I would have been the president.

Most people <u>want</u> to keep a good social life:

- I don't give up!

Tips for people living with AMD

• Being open about having AMD can help in social situations and avoid offending others, e.g., say ‘I’m sorry my eyesight’s bad but come a bit closer, onto my right side, and I'll be able to see you better’.

• If you tell others about your vision loss, most people will respond positively if you make a mistake and are happy to laugh it off. This will help avoid feelings of frustration or embarrassment. You might need to remind people though, because they can forget.

How can family, friends and carers help?

• When you approach someone with AMD it is good to introduce yourself: ‘Hi Mary, it’s Karen from next door’.

• In a group conversation, say the name of the person you are talking to because people with AMD can't see who you are looking at: ‘John, who do you think will win the football this weekend?’

• If the person with AMD wants help with recognising others, you could tell them who is in a room: ‘John is in the back of the room with his wife’, and say people's names as they approach: ‘Hi Bob’.

• For facial expressions, you could say: ‘Jill is looking sad today’ or ‘the doctor had a big smile on his face when he said that, he's having a joke’.

• Understand that the person with AMD is <u>not</u> faking it. It is normal for them to sometimes be able to see faces and sometimes not. It is also normal in AMD to have some peripheral vision (to walk around) but reduced fine vision (e.g. have problems reading, seeing faces, cooking).

• Everyone with AMD is different. Ask them to tell you how <u>their</u> vision is affected. **To get you going, see our Conversation Starter questions that you can work through together.** You can ask if they have problems seeing faces, if they make mistakes, at what distance and under what lighting conditions, how this affects their social interactions and confidence, and what they would like you to do (or not do) to help.

This Information Sheet is based on a study by researchers at the Australian National University Research School of Psychology, funded by the Australian Research Council. Quotes are adapted versions of actual quotes from AMD patients interviewed for that study. A4 Version Sept 2018.

Faces and Social Life in Age-related Macular Degeneration

An information sheet for people living with AMD,
and their family, friends and carers

AMD can make it hard to see faces which may lead to some common mistakes:

Everyone is different, but depending on lighting and distance, faces can look blurred or distorted. Because faces can be hard to see:

<u>You might not recognise people you know:</u>

- The other day I didn't even recognise my son … he was within a yard or two of me and I didn't recognise him.
- I pass by people in the street that I know <u>very</u> well.

<u>You might accidentally say hello to someone you don't know:</u>

- I went up to someone I knew very well and started having a conversation with them. They looked at me blankly, and I realised they were not who I thought they were. It was very embarrassing!
- I am more careful now and wait for others to speak first.

<u>You might use other information to help but it doesn't always work:</u>

- I look at the way people are walking, their size, their hairstyles … and then of course if they speak it's voice recognition.
- I can make some terrible mistakes, the other information helps but it is certainly not fool-proof.

<u>You might not see facial expressions:</u>

- You can't see if you've wounded someone's feelings, so you don't realise 'oh I've hurt her, I shouldn't have said that'.
- I thought my doctor was upset with me and I couldn't work it out, but my daughter said he was having a joke just to crack the ice.

<u>You might find crowds hard:</u>

- I find social situations very difficult particularly in a crowded room
- I find crowds uninteresting … if I can't see and can't place people then it's a bit of a waste for me.

Sometimes, these problems can start when your vision loss is quite mild.

People don’t understand how AMD impacts my vision:

- It’s difficult, they forget I have AMD.
- They don’t realise my vision has deteriorated.
- I worry people think I am faking. They say ‘your eyes look perfectly normal’.
- They wonder why I can’t recognise people but I can walk around ok

Other people might think I'm rude or unfriendly:

Many people with AMD worry that others think they are ignoring them on purpose.

- People are mostly kind about mistakes (they help or laugh it off), but sometimes they do take offence: When I walked past Jenny and didn't recognise her, she yelled ‘you don’t even speak to me!’

Face problems can make social situations difficult:

Some people with AMD may <u>appear passive or disengaged</u> because they cannot see who is in a room.

- My old friend Tony was at the funeral and I would have loved to talk to him, but sadly I had no idea he was there.
- I sat there [at a social function] for fully two hours not knowing who the people at the table were, and that was pretty distressing.
- I find social situations can be a bit tricky when you can’t see what other people are feeling.
- You can’t feel completely part of what’s going on.

Some people <u>withdraw or lose confidence</u>:

- I don’t socialise anymore … I would think I would hurt people’s feelings … they thought you were getting on famously and then next time you don't recognise them … it’s easier not to put yourself in that situation.
- I am more mousey now ... I go up to the social area and sit down quietly, whereas one time I would have been the president.

Most people <u>want</u> to keep a good social life:

- I don't give up!

Tips for people living with AMD

• Being open about having AMD can help in social situations and avoid offending others, e.g., say ‘I’m sorry my eyesight’s bad but come a bit closer, onto my right side, and I'll be able to see you better’.

• If you tell others about your vision loss, most people will respond positively if you make a mistake and are happy to laugh it off. This will help avoid feelings of frustration or embarrassment. You might need to remind people though, because they can forget.

How can family, friends and carers help?

• When you approach someone with AMD it is good to introduce yourself: ‘Hi Mary, it’s Karen from next door’.

• In a group conversation, say the name of the person you are talking to because people with AMD can't see who you are looking at: ‘John, who do you think will win the football this weekend?’

• If the person with AMD wants help with recognising others, you could tell them who is in a room: ‘John is in the back of the room with his wife’, and say people's names as they approach: ‘Hi Bob’.

• For facial expressions, you could say: ‘Jill is looking sad today’ or ‘the doctor had a big smile on his face when he said that, he's having a joke’.

• Understand that the person with AMD is not faking it. It is normal for them to sometimes be able to see faces and sometimes not. It is also normal in AMD to have some peripheral vision (to walk around) but reduced fine vision (e.g. have problems reading, seeing faces, cooking).

• Everyone with AMD is different. Ask them to tell you how their vision is affected. **To get you going, see our Conversation Starter questions that you can work through together.** You can ask if they have problems seeing faces, if they make mistakes, at what distance and under what lighting conditions, how this affects their social interactions and confidence, and what they would like you to do (or not do) to help.

This Information Sheet is based on a study by researchers at the Australian National University Research School of Psychology, funded by the Australian Research Council. Quotes are adapted versions of actual quotes from AMD patients interviewed for that study. US letter version 2018.

Faces and Social Life in Age-related Macular Degeneration: A Conversation Starter

The information sheet *Faces and Social Life in AMD* describes some of the difficulties with faces and social interactions that can be experienced by some people living with AMD.

Not everyone experiences the same problems. Also, the problems they experience may change as their eyesight changes.

The following questions are designed to start a conversation between a person with AMD and their family, friends, and carers (e.g., nursing home staff).

The aim is to share information about **this person's** individual experiences in seeing faces, the impact this is having on their interactions and social life, and what they would like other people to do to help.

Questions can be read out loud by the family member, friend, or carer.

<u>Seeing faces</u>

• Do you have problems seeing faces?
  - How often? Just sometimes, or almost always?

• Can you see people's faces OK if they are close by? For example, if you stand or sit next to them and are having a conversation?
  [Move to a conversational distance, i.e., 1-1.5 metres apart]
  - What does my face look like to you now?
  - Is my face clear or blurred?
  - Can you see my facial features clearly? e.g., my eyes, nose, lips?
  - Can you see who I am?
  - Can you see when I change my expressions? *[make a sad then happy face]*
  - Can you see where I'm looking? *[shift eyes to left then right]*

• Can you see people's faces OK if they are further away, like on the other side of the room?
  [Move to the other side of the room]
  - What does my face look like to you now?
  - Is my face clear or blurred?
  - Can you see my facial features clearly? e.g., my eyes, nose, lips?
  - Can you see who I am?
  - Can you see when I change my expressions? *[make a sad then happy face]*
  - Can you see where I'm looking? *[shift eyes to left then right]*

• What lighting makes it easier or harder for you to see faces?
  - Do you like strong light? Or weak light?

• Are there other things that make it easier or harder to see faces sometimes?

Social interactions

- Do you sometimes make mistakes recognising people because you can't see their face clearly?
  - Have you failed to recognise someone you know, like walking straight past a friend, or not recognising a family member?
  - Have you ever said hello to someone you thought you knew, and then it turned out it was someone else?
  - When you make one of these mistakes, do you worry about what the other person might think?
  - Do you tell them about your vision problem?

- Do you try to use other information about people to help recognise who they are, like their hairstyle, or the way they walk, or their voice?
  - How often do these things actually work, so you can tell who the person is even if you can't recognise the face?

- Have you changed your behaviour?
  - Do you tend to wait for others to speak first because you can’t recognise them?
  - Do you wait for others to get closer to you, or move yourself closer to them to work out who they are?
  - Do you smile at everyone to avoid offending people because you can’t tell whether you know them or not?

- Are you sometimes unable to see other people’s facial expressions, like whether they are looking happy, sad, angry, or bored?
  - Have you made mistakes understanding how someone is feeling, like thinking they are happy and only later realising they are sad?
  - Do you ever have no idea how others are responding to you, such as if they like you or not, or if they are enjoying your conversation or they are bored?
  - Do you sometimes fail to pick up on jokes because you can't see facial expressions?

- Do you try to use other information to help work out what people are feeling, like their tone of voice, or their body language?
  - How often do these things actually work, so you can tell how a person is feeling even if you can't see their face?

- Do you ever have trouble making eye contact with people, or telling whether someone is looking at you?

- Do you find it takes more concentration or mental effort to follow a conversation, because you can't see faces properly?

- If you lip read, do you find it hard to understand what people are saying because of problems seeing their mouth?

- Do you find it particularly hard to follow what is going on when you are in a group?
  - Why? Is it partly because sometimes you aren't sure who everyone is?
  - It is partly because you can't see everyone's expressions or where they are looking?

• Do these difficulties make it harder for you to fully engage in conversations so instead you sit quietly in social situations?

• Have problems seeing faces made social situations harder, or less rewarding?
  - Does it make it harder to join in, and to feel you are fully part of the group?
  - Does it mean you miss out sometimes, like missing out on talking to a friend because you couldn't see them at the back of the room and you didn't know they were there?
  - Are you worried others will think badly of you if you make mistakes, or accidentally ignore them?

• Do you ever just want to avoid social situations?

How would you like others to help?

[Replace 'Mary' below with actual name of the person with AMD].

• Would it be helpful if people introduce themselves when they approach, so you know who they are, like saying: *'Hi [Mary], it's Karen'* ?

• In group settings, would it be helpful if everyone says the name of the person they are speaking to each time, like: *'John, who do you think will win this weekend?'* and *'[Mary], how about you?'*

• Would it be helpful if I said the names of people to you, like: *'Here comes Bob'*, or *'David Smith is sitting at the back of the room with his wife'* ?

• Would you like me to tell you nicely if you've made a mistake, like laugh and say *'Actually that is Bob but he looks like David'*.
  - Would you want me to say this in front of the other person, or when we are alone later?

• Would you like me to tell you if someone is looking particularly emotional, like saying: *'Jill looks upset, she's crying'*. Or help you interpret people's expressions, like saying: *'I know you might think the doctor was a bit annoyed, but actually I think we was joking because he was smiling'*.

• Would you like me to explain your problems with faces to other people, so you don't have do it all the time, or so they know you don't mean to be rude if you ignore them?
  - Who would you like me to explain to?
  - Who don't you want me to say anything to?
  - Would you rather explain yourself?

• Do you have any other ideas for things I, or other people, could do to help?

• Is there anything people currently do that **doesn't** help (including me!), and you would like them to stop doing?

Supplement 2

Full vision assessment information, including rationale for ranking patients' functional vision based on best-eye BCVA

Twenty of the 21 patients underwent a full vision assessment in a clinical setting at the Australian National University (approximately 90 minutes per patient; same payment and ethics/consent arrangements as for the interview part of the study). Visual acuity was assessed monocularly using Best Corrected Visual Acuity (BCVA) and Low Contrast Visual Acuity (LCVA) using a retro-illuminated logMAR chart mounted on a stand conforming to the ETDRS standard format [1]. Other tests were used to diagnose AMD type, and stage using the Age-Related Eye Disease Study (AREDS system) [2], and to exclude other visual disorders. These included: examination of the anterior segment of the eye using slit-lamp biomicroscopy; instilling Oxybuprocaine Hydrochloride 0.4% eye drops to anesthetise the eyes to measure intraocular pressure using Goldmann applanation tonometry and to measure central corneal thickness using a Pachmate (DGH Technology Inc., Exton, PA); 10-2 frequency doubling technology (FDT) threshold using Humphrey Matrix (Carl Zeiss Meditec, Inc., Dublin, CA). After the visual field test both eyes were dilated with Tropicamide 1% and Phenylephrine 2.5% and the following tests were done: Optical Coherence Tomography (OCT) Spectralis (Heidelberg Engineering, Heidelberg, Germany) of the retina (posterior-pole) and the peripapillary retinal nerve fibre layer (pRNFL); scan was done to measure the thickness of the RNFL surrounding the optic nerve and fundus auto-fluorescence images were also acquired; Fundus photography was performed using a Canon CR-2 (Canon Inc. Medical Equipment Group, Tokyo, Japan) digital non-mydriatic camera to get an image of the fovea, the macula and the optic nerve.

Table S1 shows BVCA, LCVA, AMD type, and AREDS stage for each eye separately.

In terms of ranking (and then grouping) our patients by severity of vision loss, we used best-eye BCVA. Empirical justification for this — rather than, for example, using LCVA or acuity information from the poorer eye — was as follows.

First, consider low-contrast visual acuity (LCVA), still from the best eye. Whichever was the patients' best eye by BCVA was also their best eye by LCVA. Best-eye LCVA was extremely highly correlated with best-eye BCVA ($r = .93$), indicating no statistical potential of LCVA to explain any additional variance in functional vision. Consistent with this, Table 2a shows that best-eye LCVA correlations with everyday visual function (on the National Eye Institute Visual Function Questionnaire, NEIVFQ [3]) were no higher than best-eye BCVA correlations, for any of the full-scale NEIVFQ-25 nor the two individual items relevant to face perception (A6 and Q11); indeed, LCVA correlations were slightly lower. Further, a stepwise regression predicting NEIVFQ-25 entering BCVA first followed by LCVA showed no independent effect of LCVA (on entering LCVA, *F change* $(1, 18) =.264$ $p=.614$, with *R square change* indicating only 1.1 % of variance was explained by LCVA).

Second, consider the other eye. Recall that the other eye also has AMD, but with lower acuity. Worst-eye BCVA was largely uncorrelated with best-eye BCVA in our sample ($r = .28$), meaning there is statistical potential for worst-eye BCVA to explain additional variance in functional vision. However, analysis discounted this possibility. Table 2a shows bivariate correlations with everyday functional vision (the NEIVFQ measures) were all nonsignificant. More importantly, stepwise regression predicting NEIVFQ-25 entering BCVA first followed by LCVA showed no independent effect of LCVA (on entering LCVA, *F change* $(1, 18) =.786$ $p=.387$, with *R square change* indicating only 3.3 % of variance was explained by worse-eye acuity). Additionally, note that worst-eye acuity showed only weak correlations with psychological wellbeing measures (Table 2b).

Table 1. Detailed vision information for both eyes (bold indicates strongest eye).

Patient code (from Table 1)	Eye	Visual Acuity [1] BCVA	LCVA	Diagnosis	Visual Acuity Repeated test [2] BCVA	LCVA	AREDS Stage [4]
P1	L	6/240	6/240	Wet AMD			4
	**R**	**6/7.5**	**6/15**	**Dry AMD**			4
P2	**L**	**6/9.5**	**6/19**	**Wet AMD**	**6/12**	**6/24**	**4**
	R	6/120	6/190	End-stage AMD	6/120	6/200	4
P3	L	6/15	6/60	Dry AMD			4
	**R**	**6/12**	**6/30**	**Dry AMD**			4
P4	L	CF	<6/240	End-stage AMD			4
	**R**	**6/12**	**6/19**	**Wet AMD**			4
P5	**L**	**6/15**	**6/38**	**Wet AMD**	**6/12**	**6/19**	4
	R	6/190	<6/240	Wet AMD	6/240	<6/240[5]	4
P6	L	6/95	6/120	End-stage AMD			4
	**R**	**6/15**	**6/30**	**Wet AMD**			4
P7	**L**	**6/15**	**6/60**	**Dry AMD**			4
	R	6/95	6/240	Dry AMD			4
P8	L	CF	<6/240	Wet AMD			4
	**R**	**6/15**	**6/60**	**Early AMD**			3
P9	L	6/24	6/38	Early AMD			3
	**R**	**6/19**	**6/30**	**Wet AMD**			4
P10	L	6/30	6/60	Dry AMD			4
	**R**	**6/19**	**6/48**	**Dry AMD**			4
P11	**L**	**6/19**	**6/48**	**Wet AMD**			4
	R	6/190	<6/240	End-stage AMD			4
P12	**L**	**6/24**	**6/38**	**Early AMD**			3
	R	6/95	6/120	End-stage AMD			4
P13	**L**	**6/24**	**6/60**	**Wet AMD**	**6/24**	**6/60**	4
	R	CF	<6/240	End-stage AMD	CF	<6/240	4
P14	L	6/190	<6/240	End-stage AMD			4
	**R**	**6/38**	**6/48**	**Wet AMD**			4
P15	**L**	**6/38**	**6/60**	**Wet AMD**			4
	R	CF	<6/240	End-stage AMD			4
P16	**L**	**6/60**	**6/95**	**Dry AMD**			4
	R	6/95	6/120	Dry AMD			4
P17 [3]	L	3/60	-	Wet AMD			**-**
	**R**	**<6/60****	**-**	**Wet AMD**			-
P18	L	6/150	6/240	Dry AMD			4
	**R**	**6/75**	**6/150**	**Dry AMD**			4
P19	**L**	**6/75**	**6/120**	**Wet AMD**	**6/24**	**6/48**	**4**
	R	6/240	<6/240	End-stage AMD	6/240	<6/240	4
P20	**L**	**6/75**	**6/190**	**Wet AMD**			4
	R	HM	<6/240	End-stage AMD			4
P21	**L**	**6/190**	**<6/240**	**End-stage AMD**			4
	R	6/240	<6/240	End-stage AMD			4

Notes:

[1] BCVA = best corrected visual acuity (high contrast), LCVA = low contrast visual acuity; CF = counting fingers, HM = hand movements. LCVA results with <6/240 indicates the patient could not read all letters on the largest line of the LCVA chart. L = left eye (i.e., OS, ocular sinister), R = right eye (i.e., OD, oculus dextrus).

[2] For the 4 patients with more than 6 months between interviews, vision testing was repeated close in time to Interview 2. Note diagnosis and AREDS stage was unchanged at the second vision assessment.

[3] P17 did not have a vision assessment at ANU. Visual acuity (BCVA only) was reported by ophthalmologist.

[4] AREDS = Age-related Eye Disease Study [2]. AREDS stages are based on anatomy of the central 6mm of the retina. Stage 1 = Early AMD, small drusen. Stage 2 = Early AMD, intermediate drusen. Stage 3 = Early AMD, large drusen. Stage 4 = Active exudative AMD, CNV (choroidal neovascularisation)/Wet AMD; or End-stage Dry AMD/sub-foveal GA (geographic atrophy). For AREDS Stages 1 to 3 it is expected visual acuity would be close to normal; for Stage 4 acuity can vary from normal to <6/60 (e.g., depending on treatment).

Table 2. Correlations (*r*) between different possible acuity measures and everyday visual function and psychological wellbeing.

		**Acuity measure used as predictor**		
	**Dependent measures**	**Best-eye BCVA**	**Best-eye LCVA**	**Worst-eye BCVA**
**a. Everyday visual function**				
	NEIVFQ-25	-.47*	-.36	-.39
	NEIVFQ-25 A6	-.58**	-.55**	-.33
	NEIVFQ-25 Q11	-.48*	-.45	-.44
**b. Psychological wellbeing**				
	Anxiety (GAI)	.44*	.49*	.10
	Depression (GDS)	.12	.23	-.08
	MacDQoL	-.41	-.39	-.23

Notes:

* $p < 0.05$ (2 tailed) ** $p < .001$(2-tailed). Correlations performed with acuity scores in LogMAR. See main text Table 2 for dependent measure details. Patient P17 did not have a vision assessment; her ophthalmologist reported her BCVA was <6/60, however 6/60 or logMAR +1.0 was entered into the correlation. P17 did not have a LCVA score; a score of 6/120 or logMAR +1.3 was entered into the correlation (which is her expected LCVA score based on her BCVA score). NEIVFQ [3], GAI [4], GDS [5], MacDQoL [6].

Supplement 3
Interview 1 initial questions (asked of all participants)

1. ***Visual problems associated with AMD:***

Interviewer: The first question I am going to ask you is about the visual problems you have due to AMD. The question is: **How much does AMD affect your vision**?
You will be asked to answer this question using one of the four following options; not at all, mildly e.g., sometimes, moderately, e.g., most of the time or severely e.g., all of the time.

Interviewer: Now I would like you to think about how your vision problems have affected particular *areas of your life* *and particular everyday tasks*. **Which areas or tasks have been made harder because of AMD?**

Interviewer: Now think about how much these have affected your quality of life, that is, how good or bad you feel your life to be. For you, **Which area or task problem has MOST reduced your quality of life**? **Which has had the LEAST effect**? **What about the others in the middle?**

2. ***Problems seeing people's faces with AMD:***

Interviewer: Now I am going to ask you if you have any problems seeing people's faces. The question is: **Has AMD made it harder for you to see people's faces**? You will be asked to answer this question using one of the four following options in the same way as last time; not at all, mildly e.g., sometimes, moderately, e.g., most of the time or severely e.g., all of the time. **Can you give examples of how AMD has made it harder for you to see people's faces?**
Some prompts if needed:

- What particular problems do you have with faces? (e.g., what types of things can or can't you see in faces anymore?).
- How has this affected your interactions with other people?
- Has it affected how much you socialise with other people?

Interviewer: The next question is: **How important is seeing other people's faces to you?** To answer this question you will be asked to use one of the four following options: not important, low importance, medium importance and high importance.

Interviewer: The next questions are to find out if your problems with seeing faces has reduced your quality of life, that is reduced how good your life is. **How much have your problems with seeing faces reduced your quality of life?** (Can you give me some examples?) **How much does this upset, bother or frustrate you?** How much do your problems with seeing faces upset, bother or frustrate you compared to your problems with other visual tasks (e.g., driving, reading)?

3. ***Identity: Problems recognising other people from their faces, and psychosocial consequences:***

Interviewer: Now I'm going to ask you specifically about one particular type of task we often do with faces, which is **recognising who other people are**. This might include, for example, recognising that a person is your son, or one of friends, or someone you used to

know from work (even though you have forgotten their name, **this is not about remembering someone's name**, but whether you recognise a person by their face**). It also includes just recognising whether you have seen a person before or not**, e.g., if there is a person shopping at your supermarket today, you can tell if you have seen that person before or not (from their face, not their clothes etc.).

The question is: **Has AMD made it harder for you to recognise people from their face?** You will be asked to answer this question using one of the four following options; not at all, mildly e.g., sometimes, moderately, e.g., most of the time or severely e.g., all of the time.

<u>Interviewer</u>: You will be asked again about the importance of this task, that is: **How important is recognising people from their face to you?** You will be asked to answer that question using one of the four following options: not important, low importance, medium importance and high importance.

<u>Interviewer:</u>
• If AMD has made it hard to recognise people from their face, can you give me some examples?
• Are some people's faces easier or harder for you to recognise than others? Do you know why? (*kids? other age groups? distinguishing features? immediate family?)*
• Are there <u>situations/places</u> in which you find it easier or harder to recognise people's faces?
• Do you find you fail to recognise people you do know? Give examples [false negatives]
• Do you ever think you recognise someone who you don't actually know? Give examples [false positives]
• Do problems like these make you upset, or embarrassed, or do they bother or frustrate you? How much? Can you give some examples?
• Did it affect the other person? If so, how? (did it upset, embarrass, or annoy them)
• Have any of the problems you have talked about changed the way you deal with other people?
• Have they made you less willing to have social interactions, or to go out?
• Have any of the problems you have talked about affected your confidence?
• Overall, how much have problems in recognising other people from their face affected your quality of life?

<u>Interviewer</u>: The next questions are related to **whether you seek help with recognising other people from their face and what other strategies you might use** to do this for yourself.
If AMD has made it hard for you to recognise people's faces:

- **Do you notice people around you help you to recognise other people? In what way? e.g., do you ask for their help?** i.e., you partner whispers you the name of a person as they walk up to you, or they might say "Hi Bob....", or introduce some identifying information into the conversation.
- **If no help is available from someone else, do you have particular strategies that you use to help get around the problem?** e.g., recognising a person by the hair or the way they walk, or clothes, or their height/weight.
- **How effective do you find these strategies?**

4. ***Problems recognising other people's facial expressions, and psychosocial consequences:***

Interviewer: Now I'm going to ask you some questions about another type of task we often do with faces, which is to recognise other peoples' facial expressions and from that their emotions (i.e., what they are feeling). This includes, for example, recognising that someone is smiling or frowning, and using their facial expressions to know when someone is happy, or sad, or angry, or bored, or in pain. **Has AMD impacted your ability to see a person's facial expressions?** Again you will be asked to answer this question using one of the four following options; not at all, mildly e.g., sometimes, moderately, e.g., most of the time or severely e.g., all of the time.

Interviewer: You will be asked again about the importance of this task.
How important is it for you to be able to see a person's facial expressions? not important, low importance, medium importance, high importance?

Interviewer:
• If AMD has made it hard to see a person's facial expressions, can you give me some examples?
• Are some facial expressions easier or harder for you to see than others? Do you know why? (smiling can see flash of teeth, surprise mouth is open etc.)
• Are there situations/places in which you find it easier or harder to see facial expressions?
• Is it easier for you to see facial expressions on a person you know really well compared to a stranger? Do you know why?
• Does not being able to see a person's facial expressions make you upset, or embarrassed, or does this bother or frustrate you? How much? Can you give some examples?
• Have you had situations where not being able to see a person's facial expressions affected the other person? If so, how? (did it upset, embarrass, or annoy them)
• Have your problems seeing a person's facial expressions changed the way you deal with other people?
• Have they made you less willing to have social interactions, or to go out?
• Have any of the problems you have talked about affected your confidence?
• Overall, how much have problems in seeing a person's facial expressions affected your quality of life?

Interviewer: The next questions are related to **whether you seek help with seeing facial expressions and what other strategies you might use** to do this for yourself.
If AMD has made it hard for you to see a person's facial expressions:

- **Do you notice people around you help you to see facial expressions or to realise how someone is feeling? In what way? e.g., do you ask for their help?** i.e., you partner whispers you that Bob is looking sad, or say something aloud in conversation e.g., "Hi Bob. You are looking a bit down today".
- **If no help is available from someone else, do you have particular strategies that you use to help get around the problem?** e.g., looking for flashes of teeth to indicate smiling, listening to the tone of the person's voice, asking them how they are feeling today.
- **How effective do you find these strategies?**

*5. **Relative importance of recognising facial expression and facial identity:***

Interviewer: Which is most important to you about face recognition: recognising who people are; or recognising their expressions? You have three options:
a) recognising who they are
b) recognising their expressions
c) both are equally important.

*6. **Visual face cues to speech:***

Interviewer: Now I'm going ask you some questions about whether you think your problems with seeing faces have affected your ability to follow other people's speech, and to follow conversations.
- **Do you find it harder to follow face-to-face conversations than you did before your AMD really started affecting your vision?**
- **Do you know if your hearing itself might be a problem as well?**
- **Do you think your ability to follow face-to-face conversations has been affected more than your ability to hear people's speech well on the phone? If so why?**

(e.g., because the speech itself seems less clear or less easy to understand what words people are saying than it used to?; because I find it harder to follow their emotions?; following conversational norms: because you don't know who is going to speak next, you don't when someone is about finish talking?).

*7. **Willingness to use technology to improve face recognition:***

Interviewer: I am now going to talk about the last topic in today's interview: the use of technology to help your ability to recognise faces. We won't be able to improve your vision itself, but the idea is to try to show you faces using technology in such a way that they become a bit easier for you to recognise. This technology doesn't exist at the moment, but we are trying to develop it, and as a first step we are asking you about what you think might be most useful and practical to you.

Watching the TV
First we're going to talk about TV. **Do you find faces and their emotions hard to recognise on TV?**

The type of thing we want to try is to see whether we can make the faces on TV easier for people with AMD to see and recognise, by enhancing the picture in some way. There are various different ways we might try to change the picture to make the faces easier -- we won't try to explain the details to you now because it is very technical. We also don't know yet whether these changes to the picture would actually work (i.e., help you) -- that's what the rest of our project [1] will be about finding out.

[1] Here, the "rest of the project" refers to other studies, not included in the present article, which involved testing whether image enhancement via face caricaturing could improve patients' identity/expression recognition.

But our question at this stage is whether: **If we WERE able to make the faces on TV easier for you to recognise, how helpful would that be to you?** not at all helpful, a little bit helpful, e.g., sometimes, somewhat helpful, e.g., most of the time, very helpful, e.g., all of the time. Can you provide more information about your answer?

<u>In real life</u>

Now I'm going to talk about real life rather than TV.
Here, we are talking about:
(a) taking photos or video of real people you are talking to or seeing at the time,
(b) using some kind of device with a screen to show you those pictures so that they are bigger than in real life, and
(c) enhancing the face pictures to make them easier to see and recognise in the same way as we would be trying for TV.

Let's go through some practical examples to make it clearer what this might mean and how it might work.

A. iPad / tablet computer

[Show the iPad, held in crook of arm, with full-size face on it].
<u>Interviewer</u>: The idea is that you would have a camera on your glasses and the face pictures would be shown on the iPad, which is a little computer.
You would press a button you are holding, or is in your pocket, to tell it when you see a face that you wanted expanded up and the iPad would expand and enhance the face pictures for you to look at, which we think should make them easier to recognise. There wouldn't be any wires or noise. Hold it to one side or the other (to use your peripheral vision*; get them to try both sides*).

Do you think this sort of set up might be useful to you in everyday life? Would it work practically? If not, what's wrong with it? (Holding other things; expense)

How enthusiastic/interested would you be about trying this type of set up in your everyday life? (if we can eventually get it to work)

- Not at all interested, mildly interested, moderately interested, very interested.

B. Smartphone

[Show the smart phone with full size face, held up close-ish to participant's face so the image is large].
<u>Interviewer</u>: Using the smartphone will be similar to the iPad or tablet computer, but you use a smaller screen (phone) held closer to your eyes, rather than a bigger screen (iPad) held in the crook of your arm.

Do you think this sort of set up might be useful to you in everyday life? Would it work practically? If not, what's wrong with it? (Holding other things)

How enthusiastic/interested would you be about trying this type of set up in your everyday life? (if we can eventually get it to work)

- Not at all interested, mildly interested, moderately interested, very interested.

C. Smart Glasses
A researcher at The University of Oxford has recently developed special glasses that can be used to display pictures on the glasses themselves, without you needing to hold anything. Again, the glasses have a built-in camera, and can expand and enhance the pictures. You would press a button to control whether you want it to show you an expanded face or whether you want to switch it off so you can see through your glasses as normal.
[Show picture of smart glasses prototype].

Do you think this sort of set up might be useful to you in everyday life? Would it work practically? If not, what's wrong with it?

How enthusiastic/interested would you be about trying this type of set up in your everyday life? Not at all interested, mildly interested, moderately interested, very interested.

D. Comparison
From the options we have discussed; iPad, Smartphone, glasses, which do you think would be most practical for you? Why?

Do you currently use an iPad or smart phone device?

E. Is computer naming enough?
All of these methods use a little computer of some sort to help, but also use your brain to do/process the actual recognition of the face.
Would it be at all useful to you if a computer was able itself to work out who a person is and tell you than name somehow aloud?
e.g. say their name in your ear?
If so, would that be all you would want, or would important things still be missing for you? e.g. would it still be important to you to be able to recognise the face yourself. (NB. Wouldn't work for expression).

F. Websites e.g., *news, internet, Facebook etc.*
Like TV, would it be useful if we could enhance face pictures on the internet? e.g., new websites, Facebook etc.

G. Other suggestions
Do you have any other suggestions related to technology or a device that could help you to see faces better?

Are there any comments or questions you have about what we have discussed today?

Supplement 4

Interview 1 example of different follow-up questions to initial questions, arising from different patient responses

The way in which the participant responded to the initial interview questions was not uniform, and the follow-up questions and discussion was based on each participant's individual response. The two examples below demonstrate the richness of responses and variability across participants: in response to the same question, P9 discussed the appearance of faces to her, whereas P16 talked about the impact of his poor face recognition on social interactions, the strategies he uses to recognise others, and variability in how well he can see.

• P9 was asked '*Can you provide examples of how AMD has made it harder for you to see people's faces*?' to which she replied: '*You mean how do I perceive them*?' She continued by explaining '*Well, their features are kind of deformed, jumbled*'. The interviewer linked P9's reports of facial distortions to her previous reports of facial blur, then saying: '*That's really interesting, it's not just the blur, it's actually the way the face is configured*' to which P9 replied: '*It is, yes'*.

• Another patient (P16) was asked the same original question: '*Can you provide examples of how AMD has made it harder for you to see people's faces?*'. He replied: '*I can meet people down the street that I have known for fifty, sixty years ... they can pass me within arms-length and they speak to me and I can't see who it is'*. The interviewer followed up with '*How does that make you feel?*'. P16 said *'Not good, sometimes I, if there are people coming towards me I can pick their walk, and listen, sometimes I know their talk, you know'*. The interviewer followed up the information about non-face strategies with: *'So you can use strategies like walking and their voice, talking'* to which P16 replied *'Yes*, *the vision is different from time to time ... sometimes I can see and sometimes I can't see anything.'*

Supplement 5

Relative importance of face perception domain compared to domains currently included in the MacDQoL

In the first section of Interview 1, *before* any questions about faces had been asked (see Supplement 3), we asked patients "What areas or tasks have been made harder because of your AMD?". This question format was deliberately open ended, and early in the interview, to obtain information about what areas/tasks came to patients' minds without prior leading questions about any particular domains. Table 3 lists the full set of responses from each patient, in the order they raised each area/task. Table 4 summarises the percentage of patients spontaneously mentioning face perception (bold responses from Table 3) as compared to percentage of patients spontaneously mentioning 10 domains currently on the MacDQoL [6]. Note this table codes only for MacDQoL domains which we would reasonably be expected to be elicited by our "areas or tasks" question format: more abstract domains (e.g., 'closest personal relationships') were not mentioned by any patients but we do not take that as meaningful given the question format would not be expected to elicit these domains.

Table 3. Individual patient responses to interview question "What areas or tasks have been made harder because of your AMD?", with face-related responses highlighted.

Patient code	What areas or tasks have been made harder because of your AMD?
P1	Reading, cutting up food, cooking, eating, dressing, hair, walking
P2	Shopping, reading
P3	Reading, **identifying people at a distance**, TV, walking, computer, driving, close work e.g., knitting, home duties e.g., pouring things, cooking
P4	Making a cup of tea, walking (especially steps/changes in height), reading, knitting, TV, going to the theatre, **recognising people when walking down the street**, shopping
P5	Everything, dressing, cooking, cutting vegetables
P6	Everything, domestic duties, filing, crosswords
P7	Reading, **recognising someone across the room,** telephone numbers, gardening, sewing
P8	Walking around, reading, cooking, cleaning, driving
P9	Reading, quilting, beading
P10	Driving, **recognising people in a crowd at a distance**, reading
P11	Writing, computer, **identifying people**, driving
P12	Reading, **watching TV because can't see people very clearly**, sewing
P13	Reading, computer, gardening, sewing, **recognising people**
P14	Work (practicing pharmacy), driving, reading, working with tools e.g., machinery
P15	Reading, needle work, close work, seeing the ballet
P16	Getting around/walking, reading, shopping, driving
P17	Reading, cooking, craft, knitting, playing DVDs, ironing, driving, TV
P18	Reading, close work e.g., sewing, cooking, cutting, gardening, home duties e.g., ironing, cleaning, things with electricity, driving, crossing streets
P19	Reading, writing, knitting, using my hands
P20	**Recognising people**, looking at fine things e.g., microwave, odd jobs at home, reading, driving
P21	Painting, reading a clock, seeing colour, knitting, crocheting, tasks around home e.g., dealing with electricity/powerpoints

Table 4. Percentage of patients spontaneously mentioning face perception as compared to 10 domains currently on the MacDQoL [6].

Domain (all except faces currently included in MacDQoL)	No of patients	% of patients (*N*=21)
Interests/Free time activities (reading, TV, radio, hobbies)	20	95
Household tasks	16	76
Get out and about (foot, car, bus, train)	11	52
**Faces**	**8**	**38**
Personal affairs (letters, bills, etc.)	3	14
Shopping	3	14
Physical appearance (clothes, grooming)	2	10
Enjoy meals	1	5
Independence	0	0
Do things for others	0	0
Mishaps or lose things	0	0

Notes:

Some domains in the MacDQoL [6] provide specific examples e.g., Interests/Free time activities (reading, TV, radio, hobbies), whereas other domains e.g., household tasks, does not provide examples. We categorised patients' responses in Table 3 into the most suitable MacDQoL domain e.g., for household tasks included cooking, gardening and use of powerpoints.

Supplement 6

Interview 2 results for patient endorsement of content included in Faces and Social Life in AMD Information Sheet

Table 5 indicates the degree to which, in Interview 2, patients endorsed the inclusion of particular concepts in our *Faces and Social Life in AMD Information Sheet*. The table includes only statements for which the key information was eventually included in the Information Sheet. The final wording in the sheet may have been simplified or shortened as compared to statement listed in table.

Note Table 5 does not list all statements that patients were asked to consider. Some additional statements were excluded from the final information sheet because patient responses indicated the statements were poorly or confusingly worded (i.e., the patients couldn't understand them), or had too much information (and therefore it was not certain which part of the statement patients were endorsing). Further, some additional statements had included draft information about severity of AMD (along the lines, for example, that patients with less severe AMD would have no trouble seeing faces/expressions, or be fine seeing them close-up) which in fact turned out to be inaccurate once we had analysed the full interview data from all participants. Another observation from patients' responses was that the information sheet should be kept purely to dealing with issues related to faces and social interactions: we found that any statements including any mention of other aspects of AMD (e.g., either medical details, or other aspects of vision loss such as driving) distracted patients by making them think the information sheet was about these topics as well, or AMD more broadly, and thus should include extensive other information about AMD (which is well-covered by existing AMD public information sheets). We also included three open ended questions about whether patients had other points they thought would be good to include, but these did not elicit any additional information.

In Table 5, where total patient numbers do not equal 19 (the number who participated in Interview 2), this is because one or more patients: did not provide specific feedback about the statement (e.g., they described their own experience or were distracted by components of the question that were not relevant for present purposes, e.g., reading and driving); said they couldn't comment because they did not have personal experience regarding the statement; or were not asked the question (due to fatigue, being distracted or they did not endorse a previous relevant statement).

Table 5. Patient Endorsement of Including Particular Statements in the Information Sheet.

Statement read to patients	# patients endorsing
**Faces and social interactions**	
People living with AMD can have problems with faces. This includes recognising who other people are, and also with seeing their facial expressions.	18/18
There is a lot of variation across the visual abilities of AMD patients.... Some find bright light useful ... whereas others are sensitive to bright light. *[Full statement included additional material on other topics; patient transcripts have been used to extract specific endorsement of information about variability.]*	10/16
Some people with AMD may appear disengaged in social situations, this may be because they cannot see who is in a room.	15/15
People with AMD [1] may also be unable to see a person's facial expressions i.e., whether someone looks happy, sad or bored. Because they cannot see facial expressions, they might miss social cues. For example, someone might be looking bored but the person with AMD can't see this so they keep on talking, or a person might be just having a joke and is smiling when they say something, but the person with AMD takes it seriously.	15/16
Do you think it would be helpful to let others know that social situations can be tricky because of vision loss in AMD? *['Yes' responses]*	10/11
Where a person with AMD has trouble with faces this can lead to a variety of difficulties in social settings. For example, people may feel less confident in social situations and they may also feel frustrated or embarrassed that they cannot recognise people from their face. These feelings can impact how a person with AMD behaves in social situations which may include becoming less interactive and engaged and more passive. For example, they might wait for people to come to them rather than approaching other people in the room. It could also include waiting for other people to speak first, before they speak.	18/18
Vision loss in AMD can affect a person's confidence in social situations	18/19
**What can be done to help?**	
So, what can be done to help? First, there are some strategies a person with AMD might try out by themselves to help with recognising people. Strategies that might be helpful to try include: waiting until the other person speaks and recognising their voice; or trying to recognise the person from distinctive clothing, or their hair, or their body shape because these things don't require such fine vision as recognising their face. Be aware, however, that these strategies do not always work, e.g., the other person may not speak, or they might have changed their appearance in some way (like their hairstyle or lost weight), or they may wear similar clothing to lots of other people.	19/19
For recognising expressions, strategies that the person with AMD might find helpful to try include: listening to tone of voice, noticing body language and context e.g., is another person engaging with others or sitting alone? Be aware, however, that these strategies do not always work e.g., people may not be open about the way they feel and behave in a way that does not indicate their emotions, and vision loss in AMD will make it difficult to pick up subtle cues from the facial expressions that indicate a person's true emotions.	18/18
There are also strategies people around the person with AMD — such as family members, friends, and carers — can try when they are having trouble seeing faces. These strategies involve giving the person extra information to help them identify who people are, and what their expressions are showing; e.g., you could:<br>- Say "Hi Bob" to identify a person aloud as they approach, or provide other clues about the person such as "How did the trip to the coast with Mary go last week?"<br>- Whisper the name of a person approaching<br>- Identify out loud familiar people sitting around the room that are too far away for the person with AMD to recognise. For example, "Aunty Jo is sitting at the back of the room with her husband". This may encourage the person with AMD to move around a room and be more engaged in social situations if they know who is present.<br>- For facial expressions and mood, say "Uncle Bruce is looking down today" or "Bob is sitting on his own and he looks tired" or "Joan had a big smile on her face when she said that, I think she is having a joke with you".	19/19
**Others don't understand/ concerns about faking it**	
The fact that AMD is invisible to other people can be difficult and confusing. For example, others can see you doing some visual tasks (walking around) and they might forget you cannot do other visual tasks (read and see faces). Do you think explaining this to others might help? *['Yes' responses]*	19/19
Because of these inconsistencies across visual tasks, other people may think a person with AMD is faking their vision loss (or people with AMD might worry that others might think this). Do you think explaining this to others might help? *['Yes' responses]*	14/14

Notes: [1] Wording originally said "with moderate AMD" but patients endorsed the statement for all levels of vision loss.

Supporting/Supplementary Information References

1. Ferris FL, Freidlin V, Kassoff A, Green SB, Milton RC. Relative letter and position difficulty on visual acuity charts from the Early Treatment Diabetic Retinopathy Study. Am J Ophthalmol. 1993;116(6): 735–740. doi: 10.1016/S0002-9394(14)73474-9
2. Age-Related Eye Disease Study (AREDS) Research Group. The Age-Related Eye Disease Study system for classifying age-related macular degeneration from stereoscopic color fundus photographs: the Age-Related Eye Disease Study Report Number 6. Am J Ophthalmol. 2001;132(5): 668-681.
3. Mangione CM, Lee PP, Gutierrez PR, Spritzer K, Berry S, Hays RD. Development of the 25-Item National Eye Institute Visual Function Questionnaire. Arch Ophthalmol. 2001;119(7): 1050-1058. doi: 10.1001/archopht.119.7.1050
4. Pachana NA, Byrne GJ, Siddle H, Koloski N, Harley E, Arnold E. Development and validation of the Geriatric Anxiety Inventory. Int Psychogeriatr. 2007;19(1): 103-14. doi: 10.1017/S1041610206003504
5. Sheikh JI, Yesavage JA. Geriatric Depression Scale (GDS): Recent evidence and development of a shorter version. Clin Gerontol. 1986;5: 165-173.
6. Mitchell J, Bradley C. Design of an individualised measure of the impact of macular disease on quality of life (the MacDQoL). Qual Life Res. 2004;13: 1163-1175.
