# Supplementary material for: Impacts of impaired face perception on social interactions and quality of life in age-related macular degeneration: A qualitative study and new community resources
Source: PLoS One. 2018 Dec 31;13(12):e0209218. doi: 10.1371/journal.pone.0209218 (PMC6312296; doi:10.1371/journal.pone.0209218)
Supplement: S2 File — This brochure is formatted to print and fold in tri-fold, size A4. Note: The guardian of the child whose image is in the brochure has given written informed consent to have their child’s image published under a CC-BY license. The child whose image is in the brochure was not a participant in the study. (PDF) [file pone.0209218.s002.pdf]

Other people might not understand you can’t see faces well.

You might worry they think you are being rude or unfriendly.

Social situations can be difficult and you might:

- Feel like you are missing out or can’t join in
- Feel less confident
- Feel tempted to avoid social situations, or experience isolation

If you want to know more

- Look at our information sheet that describes real-life experiences of people living with AMD
- Get our conversation starter. This helps people with AMD and their family, friends and carers learn more about their specific problems with seeing faces and what others can do to help.
- **Get these from**:

Lane, J. (2018) article in PLOS ONE (free access)

Faces and social life in age-related macular degeneration

AMD can make it hard to see faces

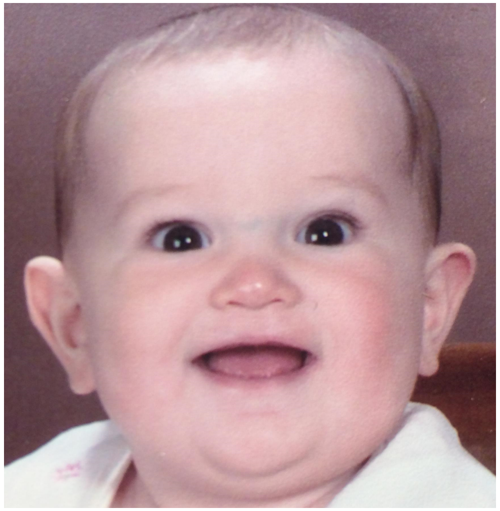

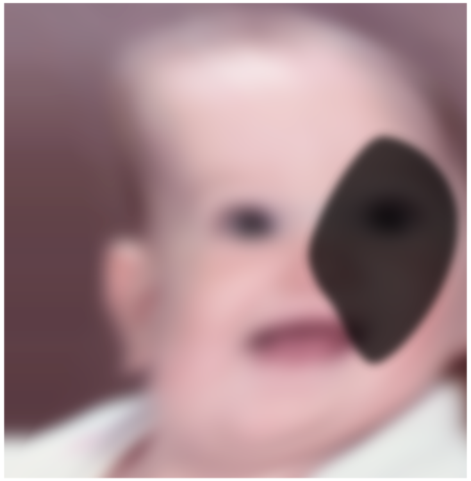

When it is hard to see faces you might make some common mistakes including:

- Ignoring someone you know, when you don't recognise them
- Accidentally saying hi to people you don't know
- Misreading facial expressions and emotions

You might also find crowds hard because you can't see who is in a room or who is talking.

Here are some tips for people living with AMD that might help:

- Tell others you can't see faces well
- Ask people to move closer so you can see their faces better
- Ask people to tell you where other people are in the room and what their facial expressions are

This brochure is based on a study by researchers at the ANU Research School of Psychology

Australian National University

How can family, friends and carers help?

- Ask if the person with AMD has problems seeing faces
- Say your name when you walk in the room or the name of other people in the room
- Understand the person with AMD is not faking their vision loss

For further information on AMD please go to: www.mdfoundation.com.au
