# Supplementary material for: Impacts of impaired face perception on social interactions and quality of life in age-related macular degeneration: A qualitative study and new community resources
Source: PLoS One. 2018 Dec 31;13(12):e0209218. doi: 10.1371/journal.pone.0209218 (PMC6312296; doi:10.1371/journal.pone.0209218)
Supplement: S4 File — This includes the rationale for ranking patients' functional vision based on best-eye BCVA and Supplementary Table A and Table B. (PDF) [file pone.0209218.s004.pdf]

Impacts of impaired face perception on social interactions and quality of life in age-related macular degeneration: A qualitative study and new community resources

Jo Lane[1], Emilie M. F. Rohan[2], Faran Sabeti[2,7], Rohan W. Essex[3], Ted Maddess[2], Amy Dawel[1], Rachel A. Robbins[4], Nick Barnes[5], Xuming He[6], Elinor McKone[1*]

PLoS ONE 20 December 2018

Full vision assessment information, including rationale for ranking patients' functional vision based on best-eye BCVA (Includes Tables A & B).

Twenty of the 21 patients underwent a full vision assessment in a clinical setting at the Australian National University (approximately 90 minutes per patient; same payment and ethics/consent arrangements as for the interview part of the study). Visual acuity was assessed monocularly using Best Corrected Visual Acuity (BCVA) and Low Contrast Visual Acuity (LCVA) using a retro-illuminated logMAR chart mounted on a stand conforming to the ETDRS standard format [1]. Other tests were used to diagnose AMD type, and stage using the Age-Related Eye Disease Study (AREDS system) [2], and to exclude other visual disorders. These included: examination of the anterior segment of the eye using slit-lamp biomicroscopy; instilling Oxybuprocaine Hydrochloride 0.4% eye drops to anesthetise the eyes to measure intraocular pressure using Goldmann applanation tonometry and to measure central corneal thickness using a Pachmate (DGH Technology Inc., Exton, PA); 10-2

frequency doubling technology (FDT) threshold using Humphrey Matrix (Carl Zeiss Meditec, Inc., Dublin, CA). After the visual field test both eyes were dilated with Tropicamide 1% and Phenylephrine 2.5% and the following tests were done: Optical Coherence Tomography (OCT) Spectralis (Heidelberg Engineering, Heidelberg, Germany) of the retina (posterior-pole) and the peripapillary retinal nerve fibre layer (pRNFL); scan was done to measure the thickness of the RNFL surrounding the optic nerve and fundus auto-fluorescence images were also acquired; Fundus photography was performed using a Canon CR-2 (Canon Inc. Medical Equipment Group, Tokyo, Japan) digital non-mydriatic camera to get an image of the fovea, the macula and the optic nerve.

Table A shows BVCA, LCVA, AMD type, and AREDS stage for each eye separately.

In terms of ranking (and then grouping) our patients by severity of vision loss, we used best-eye BCVA. Empirical justification for this — rather than, for example, using LCVA or acuity information from the poorer eye — was as follows.

First, consider low-contrast visual acuity (LCVA), still from the best eye. Whichever was the patients' best eye by BCVA was also their best eye by LCVA. Best-eye LCVA was extremely highly correlated with best-eye BCVA (r = .93), indicating no statistical potential of LCVA to explain any additional variance in functional vision. Consistent with this, Table B (top half) shows that best-eye LCVA correlations with everyday visual function (on the National Eye Institute Visual Function Questionnaire, NEIVFQ [3]) were no higher than best-eye BCVA correlations, for any of the full-scale NEIVFQ-25 nor the two individual items relevant to face perception (A6 and Q11); indeed, LCVA correlations were slightly lower. Further, a stepwise regression predicting NEIVFQ-25 entering BCVA first followed by LCVA showed no independent effect of LCVA (on entering LCVA, *F change* (1, 18) =.264 *p*=.614, with *R square change* indicating only 1.1 % of variance was explained by LCVA).

Second, consider the other eye. Recall that the other eye also has AMD, but with lower acuity. Worst-eye BCVA was largely uncorrelated with best-eye BCVA in our sample (r = .28), meaning there is statistical potential for worst-eye BCVA to explain additional variance in functional vision. However, analysis discounted this possibility. Table B (top half) shows bivariate correlations with everyday functional vision (the NEIVFQ measures) were all nonsignificant. More importantly, stepwise regression predicting NEIVFQ-25 entering BCVA first followed by LCVA showed no independent effect of LCVA (on entering LCVA, *F change* (1, 18) =.786 p=.387, with *R square change* indicating only 3.3 % of variance was explained by worse-eye acuity). Additionally, note that worst-eye acuity showed only weak correlations with psychological wellbeing measures (Table B bottom half).

Table A. Detailed vision information for both eyes (bold indicates strongest eye).

Patient code (from Table 1)	Eye	Visual Acuity [1]		Diagnosis	Visual Acuity Repeated test [2]		AREDS Stage [4]
		BCVA	LCVA		BCVA	LCVA	
P1	L	6/240	6/240	Wet AMD			4
	**R**	**6/7.5**	**6/15**	**Dry AMD**			4
P2	**L**	**6/9.5**	**6/19**	**Wet AMD**	**6/12**	**6/24**	**4**
	R	6/120	6/190	End-stage AMD	6/120	6/200	4
P3	L	6/15	6/60	Dry AMD			4
	**R**	**6/12**	**6/30**	**Dry AMD**			4
P4	L	CF	<6/240	End-stage AMD			4
	**R**	**6/12**	**6/19**	**Wet AMD**			4
P5	**L**	**6/15**	**6/38**	**Wet AMD**	**6/12**	**6/19**	4
	R	6/190	<6/240	Wet AMD	6/240	<6/240[5]	4
P6	L	6/95	6/120	End-stage AMD			4
	**R**	**6/15**	**6/30**	**Wet AMD**			4
P7	**L**	**6/15**	**6/60**	**Dry AMD**			4
	R	6/95	6/240	Dry AMD			4
P8	L	CF	<6/240	Wet AMD			4
	**R**	**6/15**	**6/60**	**Early AMD**			3
P9	L	6/24	6/38	Early AMD			3
	**R**	**6/19**	**6/30**	**Wet AMD**			4
P10	L	6/30	6/60	Dry AMD			4
	**R**	**6/19**	**6/48**	**Dry AMD**			4
P11	**L**	**6/19**	**6/48**	**Wet AMD**			4
	R	6/190	<6/240	End-stage AMD			4
P12	**L**	**6/24**	**6/38**	**Early AMD**			3
	R	6/95	6/120	End-stage AMD			4
P13	**L**	**6/24**	**6/60**	**Wet AMD**	**6/24**	**6/60**	4
	R	CF	<6/240	End-stage AMD	CF	<6/240	4
P14	L	6/190	<6/240	End-stage AMD			4
	**R**	**6/38**	**6/48**	**Wet AMD**			4
P15	**L**	**6/38**	**6/60**	**Wet AMD**			4
	R	CF	<6/240	End-stage AMD			4
P16	**L**	**6/60**	**6/95**	**Dry AMD**			4
	R	6/95	6/120	Dry AMD			4
P17 [3]	L	3/60	-	Wet AMD			-
	**R**	**<6/60****	**-**	**Wet AMD**			-
P18	L	6/150	6/240	Dry AMD			4
	**R**	**6/75**	**6/150**	**Dry AMD**			4
P19	**L**	**6/75**	**6/120**	**Wet AMD**	**6/24**	**6/48**	**4**
	R	6/240	<6/240	End-stage AMD	6/240	<6/240	4
P20	**L**	**6/75**	**6/190**	**Wet AMD**			4
	R	HM	<6/240	End-stage AMD			4
P21	**L**	**6/190**	**<6/240**	**End-stage AMD**			4
	R	6/240	<6/240	End-stage AMD			4

Notes:

[1] BCVA = best corrected visual acuity (high contrast), LCVA = low contrast visual acuity; CF = counting fingers, HM = hand movements. LCVA results with <6/240 indicates the patient could not read all letters on the largest line of the LCVA chart. L = left eye (i.e., OS, ocular sinister), R = right eye (i.e., OD, oculus dextrus).

[2] For the 4 patients with more than 6 months between interviews, vision testing was repeated close in time to Interview 2. Note diagnosis and AREDS stage was unchanged at the second vision assessment.

[3] P17 did not have a vision assessment at ANU. Visual acuity (BCVA only) was reported by ophthalmologist.

[4] AREDS = Age-related Eye Disease Study [2]. AREDS stages are based on anatomy of the central 6mm of the retina. Stage 1 = Early AMD, small drusen. Stage 2 = Early AMD, intermediate drusen. Stage 3 = Early AMD, large drusen. Stage 4 = Active exudative AMD, CNV (choroidal neovascularisation)/Wet AMD; or End-stage Dry AMD/sub-foveal GA (geographic atrophy). For AREDS Stages 1 to 3 it is expected visual acuity would be close to normal; for Stage 4 acuity can vary from normal to <6/60 (e.g., depending on treatment).

Table B. Correlations (*r*) between different possible acuity measures and everyday visual function and psychological wellbeing.

		Acuity measure used as predictor		
	Dependent measures	Best-eye BCVA	Best-eye LCVA	Worst-eye BCVA
**Everyday visual function**				
	NEIVFQ-25	-.47*	-.36	-.39
	NEIVFQ-25 A6	-.58**	-.55**	-.33
	NEIVFQ-25 Q11	-.48*	-.45	-.44
**Psychological wellbeing**				
	Anxiety (GAI)	.44*	.49*	.10
	Depression (GDS)	.12	.23	-.08
	MacDQoL	-.41	-.39	-.23

Notes:
* $p < 0.05$ (2 tailed) ** $p < .001$(2-tailed). Correlations performed with acuity scores in LogMAR. See main text Table 2 for dependent measure details. Patient P17 did not have a vision assessment; her ophthalmologist reported her BCVA was <6/60, however 6/60 or logMAR +1.0 was entered into the correlation. P17 did not have a LCVA score; a score of 6/120 or logMAR +1.3 was entered into the correlation (which is her expected LCVA score based on her BCVA score). NEIVFQ [3], GAI [4], GDS [5], MacDQoL [6].

References for S4 File

1. Ferris FL, Freidlin V, Kassoff A, Green SB, Milton RC. Relative letter and position difficulty on visual acuity charts from the Early Treatment Diabetic Retinopathy Study. Am J Ophthalmol. 1993;116(6): 735–740. doi: 10.1016/S0002-9394(14)73474-9
2. Age-Related Eye Disease Study (AREDS) Research Group. The Age-Related Eye Disease Study system for classifying age-related macular degeneration from stereoscopic color fundus photographs: the Age-Related Eye Disease Study Report Number 6. Am J Ophthalmol. 2001;132(5): 668-681.
3. Mangione CM, Lee PP, Gutierrez PR, Spritzer K, Berry S, Hays RD. Development of the 25-Item National Eye Institute Visual Function Questionnaire. Arch Ophthalmol. 2001;119(7): 1050-1058. doi: 10.1001/archopht.119.7.1050

4. Pachana NA, Byrne GJ, Siddle H, Koloski N, Harley E, Arnold E. Development and validation of the Geriatric Anxiety Inventory. Int Psychogeriatr. 2007;19(1): 103-14. doi: 10.1017/S1041610206003504

5. Sheikh JI, Yesavage JA. Geriatric Depression Scale (GDS): Recent evidence and development of a shorter version. Clin Gerontol. 1986;5: 165-173.

6. Mitchell J, Bradley C. Design of an individualised measure of the impact of macular disease on quality of life (the MacDQoL). Qual Life Res. 2004;13: 1163-1175.
