# Supplementary material for: Impacts of impaired face perception on social interactions and quality of life in age-related macular degeneration: A qualitative study and new community resources
Source: PLoS One. 2018 Dec 31;13(12):e0209218. doi: 10.1371/journal.pone.0209218 (PMC6312296; doi:10.1371/journal.pone.0209218)
Supplement: S5 File — (PDF) [file pone.0209218.s005.pdf]

Impacts of impaired face perception on social interactions and quality of life in age-related macular degeneration: A qualitative study and new community resources

Jo Lane[1], Emilie M. F. Rohan[2], Faran Sabeti[2,7], Rohan W. Essex[3], Ted Maddess[2], Amy Dawel[1], Rachel A. Robbins[4], Nick Barnes[5], Xuming He[6], Elinor McKone[1*]

PLoS ONE 20 December 2018

Interview 1 initial questions (asked of all participants)

1. Visual problems associated with AMD:

Interviewer: The first question I am going to ask you is about the visual problems you have due to AMD. The question is: **How much does AMD affect your vision**?
You will be asked to answer this question using one of the four following options; not at all, mildly e.g., sometimes, moderately, e.g., most of the time or severely e.g., all of the time.

Interviewer: Now I would like you to think about how your vision problems have affected particular *areas of your life* *and particular everyday tasks*. **Which areas or tasks have been made harder because of AMD?**

Interviewer: Now think about how much these have affected your quality of life, that is, how good or bad you feel your life to be. For you, **Which area or task problem has MOST reduced your quality of life**? **Which has had the LEAST effect**? **What about the others in the middle?**

2. Problems seeing people's faces with AMD:

Interviewer: Now I am going to ask you if you have any problems seeing people's faces. The question is: **Has AMD made it harder for you to see people's faces**? You will be asked to answer this question using one of the four following options in the same way as last time; not at all, mildly e.g., sometimes, moderately, e.g., most of the time or severely e.g., all of the time. **Can you give examples of how AMD has made it harder for you to see people's faces?**
Some prompts if needed:

• What particular problems do you have with faces? (e.g., what types of things can or can't you see in faces anymore?).

- How has this affected your interactions with other people?
- Has it affected how much you socialise with other people?

Interviewer: The next question is: **How important is seeing other people's faces to you?** To answer this question you will be asked to use one of the four following options: not important, low importance, medium importance and high importance.

Interviewer: The next questions are to find out if your problems with seeing faces has reduced your quality of life, that is reduced how good your life is. **How much have your problems with seeing faces reduced your quality of life?** (Can you give me some examples?) **How much does this upset, bother or frustrate you?** How much do your problems with seeing faces upset, bother or frustrate you compared to your problems with other visual tasks (e.g., driving, reading)?

3. ***Identity: Problems recognising other people from their faces, and psychosocial consequences:***

Interviewer: Now I'm going to ask you specifically about one particular type of task we often do with faces, which is **recognising who other people are**. This might include, for example, recognising that a person is your son, or one of friends, or someone you used to know from work (even though you have forgotten their name, **this is not about remembering someone's name**, but whether you recognise a person by their face**). It also includes just recognising whether you have seen a person before or not**, e.g., if there is a person shopping at your supermarket today, you can tell if you have seen that person before or not (from their face, not their clothes etc.).

The question is: **Has AMD made it harder for you to recognise people from their face?** You will be asked to answer this question using one of the four following options; not at all, mildly e.g., sometimes, moderately, e.g., most of the time or severely e.g., all of the time.

Interviewer: You will be asked again about the importance of this task, that is: **How important is recognising people from their face to you?** You will be asked to answer that question using one of the four following options: not important, low importance, medium importance and high importance.

Interviewer:

- **If AMD has made it hard to recognise people from their face, can you give me some examples?**
- **Are some people's faces easier or harder for you to recognise than others? Do you know why?** (*kids? other age groups? distinguishing features? immediate family?)*
- **Are there situations/places in which you find it easier or harder to recognise people's faces?**
- **Do you find you fail to recognise people you do know?** Give examples [false negatives]
- **Do you ever think you recognise someone who you don't actually know?** Give examples [false positives]
- **Do problems like these make you upset, or embarrassed, or do they bother or frustrate you?** How much? Can you give some examples?
- **Did it affect the other person? If so, how?** (did it upset, embarrass, or annoy them)
- **Have any of the problems you have talked about changed the way you deal with other people?**

• Have they made you less willing to have social interactions, or to go out?
• Have any of the problems you have talked about affected your confidence?
• Overall, how much have problems in recognising other people from their face affected your quality of life?

Interviewer: The next questions are related to **whether you seek help with recognising other people from their face and what other strategies you might use** to do this for yourself.
If AMD has made it hard for you to recognise people's faces:

- **Do you notice people around you help you to recognise other people? In what way? e.g., do you ask for their help?** i.e., you partner whispers you the name of a person as they walk up to you, or they might say "Hi Bob....", or introduce some identifying information into the conversation.
- **If no help is available from someone else, do you have particular strategies that you use to help get around the problem?** e.g., recognising a person by the hair or the way they walk, or clothes, or their height/weight.
- **How effective do you find these strategies?**

4. *Problems recognising other people's facial expressions, and psychosocial consequences:*

Interviewer: Now I'm going to ask you some questions about another type of task we often do with faces, which is to recognise other peoples' facial expressions and from that their emotions (i.e., what they are feeling). This includes, for example, recognising that someone is smiling or frowning, and using their facial expressions to know when someone is happy, or sad, or angry, or bored, or in pain. **Has AMD impacted your ability to see a person's facial expressions?** Again you will be asked to answer this question using one of the four following options; not at all, mildly e.g., sometimes, moderately, e.g., most of the time or severely e.g., all of the time.

Interviewer: You will be asked again about the importance of this task.
How important is it for you to be able to see a person's facial expressions? not important, low importance, medium importance, high importance?

Interviewer:
• If AMD has made it hard to see a person's facial expressions, can you give me some examples?
• Are some facial expressions easier or harder for you to see than others? Do you know why? (smiling can see flash of teeth, surprise mouth is open etc.)
• Are there situations/places in which you find it easier or harder to see facial expressions?
• Is it easier for you to see facial expressions on a person you know really well compared to a stranger? Do you know why?
• Does not being able to see a person's facial expressions make you upset, or embarrassed, or does this bother or frustrate you? How much? Can you give some examples?
• Have you had situations where not being able to see a person's facial expressions affected the other person? If so, how? (did it upset, embarrass, or annoy them)

• Have your problems seeing a person's facial expressions changed the way you deal with other people?
• Have they made you less willing to have social interactions, or to go out?
• Have any of the problems you have talked about affected your confidence?
• Overall, how much have problems in seeing a person's facial expressions affected your quality of life?

Interviewer: The next questions are related to **whether you seek help with seeing facial expressions and what other strategies you might use** to do this for yourself.
If AMD has made it hard for you to see a person's facial expressions:

- **Do you notice people around you help you to see facial expressions or to realise how someone is feeling? In what way? e.g., do you ask for their help?** i.e., you partner whispers you that Bob is looking sad, or say something aloud in conversation e.g., "Hi Bob. You are looking a bit down today".
- **If no help is available from someone else, do you have particular strategies that you use to help get around the problem?** e.g., looking for flashes of teeth to indicate smiling, listening to the tone of the person's voice, asking them how they are feeling today.
- **How effective do you find these strategies?**

5. Relative importance of recognising facial expression and facial identity:

Interviewer: Which is most important to you about face recognition: recognising who people are; or recognising their expressions? You have three options:
a) recognising who they are
b) recognising their expressions
c) both are equally important.

6. Visual face cues to speech:

Interviewer: Now I'm going ask you some questions about whether you think your problems with seeing faces have affected your ability to follow other people's speech, and to follow conversations.
• **Do you find it harder to follow face-to-face conversations than you did before your AMD really started affecting your vision?**
• **Do you know if your hearing itself might be a problem as well?**
• **Do you think your ability to follow face-to-face conversations has been affected more than your ability to hear people's speech well on the phone? If so why?**
(e.g., because the speech itself seems less clear or less easy to understand what words people are saying than it used to?; because I find it harder to follow their emotions?; following conversational norms: because you don't know who is going to speak next, you don't when someone is about finish talking?).

7. Willingness to use technology to improve face recognition:

Interviewer: I am now going to talk about the last topic in today's interview: the use of technology to help your ability to recognise faces. We won't be able to improve your vision itself, but the idea is to try to show you faces using technology in such a way that they become a bit easier for you to recognise. This technology doesn't exist at the moment, but we are trying to develop it, and as a first step we are asking you about what you think might be most useful and practical to you.

Watching the TV
First we're going to talk about TV. **Do you find faces and their emotions hard to recognise on TV?**

The type of thing we want to try is to see whether we can make the faces on TV easier for people with AMD to see and recognise, by enhancing the picture in some way. There are various different ways we might try to change the picture to make the faces easier -- we won't try to explain the details to you now because it is very technical. We also don't know yet whether these changes to the picture would actually work (i.e., help you) -- that's what the rest of our project [1] will be about finding out.

[1] Here, the "rest of the project" refers to other studies, not included in the present article, which involved testing whether image enhancement via face caricaturing could improve patients' identity/expression recognition.

But our question at this stage is whether: **If we WERE able to make the faces on TV easier for you to recognise, how helpful would that be to you?** not at all helpful, a little bit helpful, e.g., sometimes, somewhat helpful, e.g., most of the time, very helpful, e.g., all of the time. Can you provide more information about your answer?

<u>In real life</u>
Now I'm going to talk about real life rather than TV.
Here, we are talking about:
(a) taking photos or video of real people you are talking to or seeing at the time,
(b) using some kind of device with a screen to show you those pictures so that they are bigger than in real life, and
(c) enhancing the face pictures to make them easier to see and recognise in the same way as we would be trying for TV.

Let's go through some practical examples to make it clearer what this might mean and how it might work.

A. iPad / tablet computer
[Show the iPad, held in crook of arm, with full-size face on it].
<u>Interviewer</u>: The idea is that you would have a camera on your glasses and the face pictures would be shown on the iPad, which is a little computer.
You would press a button you are holding, or is in your pocket, to tell it when you see a face that you wanted expanded up and the iPad would expand and enhance the face pictures for you to look at, which we think should make them easier to recognise. There wouldn't be any wires or noise. Hold it to one side or the other (to use your peripheral vision*; get them to try both sides*).

Do you think this sort of set up might be useful to you in everyday life? Would it work practically? If not, what's wrong with it? (Holding other things; expense)

How enthusiastic/interested would you be about trying this type of set up in your everyday life? (if we can eventually get it to work)

- Not at all interested, mildly interested, moderately interested, very interested.

B. Smartphone
[Show the smart phone with full size face, held up close-ish to participant's face so the image is large].
<u>Interviewer</u>: Using the smartphone will be similar to the iPad or tablet computer, but you use a smaller screen (phone) held closer to your eyes, rather than a bigger screen (iPad) held in the crook of your arm.

Do you think this sort of set up might be useful to you in everyday life? Would it work practically? If not, what's wrong with it? (Holding other things)

How enthusiastic/interested would you be about trying this type of set up in your everyday life? (if we can eventually get it to work)

- Not at all interested, mildly interested, moderately interested, very interested.

C. Smart Glasses
A researcher at The University of Oxford has recently developed special glasses that can be used to display pictures on the glasses themselves, without you needing to hold anything. Again, the glasses have a built-in camera, and can expand and enhance the pictures. You would press a button to control whether you want it to show you an expanded face or whether you want to switch it off so you can see through your glasses as normal.
[Show picture of smart glasses prototype].

Do you think this sort of set up might be useful to you in everyday life? Would it work practically? If not, what's wrong with it?

How enthusiastic/interested would you be about trying this type of set up in your everyday life? Not at all interested, mildly interested, moderately interested, very interested.

D. Comparison
From the options we have discussed; iPad, Smartphone, glasses, which do you think would be most practical for you? Why?

Do you currently use an iPad or smart phone device?

E. Is computer naming enough?
All of these methods use a little computer of some sort to help, but also use your brain to do/process the actual recognition of the face.
Would it be at all useful to you if a computer was able itself to work out who a person is and tell you than name somehow aloud?
e.g. say their name in your ear?
If so, would that be all you would want, or would important things still be missing for you? e.g. would it still be important to you to be able to recognise the face yourself. (NB. Wouldn't work for expression).

F. Websites e.g., news, internet, Facebook etc.
Like TV, would it be useful if we could enhance face pictures on the internet? e.g., new websites, Facebook etc.

G. Other suggestions
Do you have any other suggestions related to technology or a device that could help you to see faces better?

Are there any comments or questions you have about what we have discussed today?
