# Supplementary material for: Impacts of impaired face perception on social interactions and quality of life in age-related macular degeneration: A qualitative study and new community resources
Source: PLoS One. 2018 Dec 31;13(12):e0209218. doi: 10.1371/journal.pone.0209218 (PMC6312296; doi:10.1371/journal.pone.0209218)
Supplement: S6 File — (PDF) [file pone.0209218.s006.pdf]

Impacts of impaired face perception on social interactions and quality of life in age-related macular degeneration: A qualitative study and new community resources

Jo Lane[1], Emilie M. F. Rohan[2], Faran Sabeti[2,7], Rohan W. Essex[3], Ted Maddess[2],
Amy Dawel[1], Rachel A. Robbins[4], Nick Barnes[5], Xuming He[6], Elinor McKone[1*]

PLoS ONE 20 December 2018

Interview 1 example of different follow-up questions to initial questions, arising from different patient responses

The way in which the participant responded to the initial interview questions was not uniform, and the follow-up questions and discussion was based on each participant's individual response. The two examples below demonstrate the richness of responses and variability across participants: in response to the same question, P9 discussed the appearance of faces to her, whereas P16 talked about the impact of his poor face recognition on social interactions, the strategies he uses to recognise others, and variability in how well he can see.

• P9 was asked '*Can you provide examples of how AMD has made it harder for you to see people's faces*?' to which she replied: '*You mean how do I perceive them*?' She continued by explaining '*Well, their features are kind of deformed, jumbled*'. The interviewer linked P9's reports of facial distortions to her previous reports of facial blur, then saying: '*That's really interesting, it's not just the blur, it's actually the way the face is configured*' to which P9 replied: '*It is, yes'*.

• Another patient (P16) was asked the same original question: '*Can you provide examples of how AMD has made it harder for you to see people's faces?*'. He replied: '*I can meet people down the street that I have known for fifty, sixty years … they can pass me within arms-length and they speak to me and I can't see who it is'*. The interviewer followed up with '*How does that make you feel?*'. P16 said *'Not good, sometimes I, if there are people coming towards me I can pick their walk, and listen, sometimes I know their talk, you know'*. The interviewer followed up the information about non-face strategies with: *'So you can use strategies like walking and their voice, talking'* to which P16 replied *'Yes*, *the vision is different from time to time … sometimes I can see and sometimes I can't see anything.'*
