# Supplementary material for: Impacts of impaired face perception on social interactions and quality of life in age-related macular degeneration: A qualitative study and new community resources
Source: PLoS One. 2018 Dec 31;13(12):e0209218. doi: 10.1371/journal.pone.0209218 (PMC6312296; doi:10.1371/journal.pone.0209218)
Supplement: S7 File — Includes Supplementary Table A and B. (PDF) [file pone.0209218.s007.pdf]

Impacts of impaired face perception on social interactions and quality of life in age-related macular degeneration: A qualitative study and new community resources

Jo Lane[1], Emilie M. F. Rohan[2], Faran Sabeti[2,7], Rohan W. Essex[3], Ted Maddess[2], Amy Dawel[1], Rachel A. Robbins[4], Nick Barnes[5], Xuming He[6], Elinor McKone[1*]

PLoS ONE 20 December 2018

Relative importance of face perception domain compared to domains currently included in the MacDQoL (Includes Tables A & B).

In the first section of Interview 1, *before* any questions about faces had been asked (see Supplement 3), we asked patients “What areas or tasks have been made harder because of your AMD?”. This question format was deliberately open ended, and early in the interview, to obtain information about what areas/tasks came to patients' minds without prior leading questions about any particular domains. Table A lists the full set of responses from each patient, in the order they raised each area/task. Table B summarises the percentage of patients spontaneously mentioning face perception (bold responses from Table A) as compared to percentage of patients spontaneously mentioning 10 domains currently on the MacDQoL [1]. Note this table codes only for MacDQoL domains which we would reasonably be expected to be elicited by our "areas or tasks" question format: more abstract domains (e.g., 'closest personal relationships') were not mentioned by any patients but we do not take that as meaningful given the question format would not be expected to elicit these domains.

Table A. Individual patient responses to interview question "What areas or tasks have been made harder because of your AMD?", with face-related responses highlighted.

Patient code	What areas or tasks have been made harder because of your AMD?
P1	Reading, cutting up food, cooking, eating, dressing, hair, walking
P2	Shopping, reading
P3	Reading, **identifying people at a distance**, TV, walking, computer, driving, close work e.g., knitting, home duties e.g., pouring things, cooking
P4	Making a cup of tea, walking (especially steps/changes in height), reading, knitting, TV, going to the theatre, **recognising people when walking down the street**, shopping
P5	Everything, dressing, cooking, cutting vegetables
P6	Everything, domestic duties, filing, crosswords
P7	Reading, **recognising someone across the room,** telephone numbers, gardening, sewing
P8	Walking around, reading, cooking, cleaning, driving
P9	Reading, quilting, beading
P10	Driving, **recognising people in a crowd at a distance**, reading
P11	Writing, computer, **identifying people**, driving
P12	Reading, **watching TV because can't see people very clearly**, sewing
P13	Reading, computer, gardening, sewing, **recognising people**
P14	Work (practicing pharmacy), driving, reading, working with tools e.g., machinery
P15	Reading, needle work, close work, seeing the ballet
P16	Getting around/walking, reading, shopping, driving
P17	Reading, cooking, craft, knitting, playing DVDs, ironing, driving, TV
P18	Reading, close work e.g., sewing, cooking, cutting, gardening, home duties e.g., ironing, cleaning, things with electricity, driving, crossing streets
P19	Reading, writing, knitting, using my hands
P20	**Recognising people**, looking at fine things e.g., microwave, odd jobs at home, reading, driving
P21	Painting, reading a clock, seeing colour, knitting, crocheting, tasks around home e.g., dealing with electricity/powerpoints

Table B. Percentage of patients spontaneously mentioning face perception as compared to 10 domains currently on the MacDQoL [1].

Domain (all except faces currently included in MacDQoL)	No of patients	% of patients (*N*=21)
Interests/Free time activities (reading, TV, radio, hobbies)	20	95
Household tasks	16	76
Get out and about (foot, car, bus, train)	11	52
**Faces**	**8**	**38**
Personal affairs (letters, bills, etc.)	3	14
Shopping	3	14
Physical appearance (clothes, grooming)	2	10
Enjoy meals	1	5
Independence	0	0
Do things for others	0	0
Mishaps or lose things	0	0

Notes:

Some domains in the MacDQoL [1] provide specific examples e.g., Interests/Free time activities (reading, TV, radio, hobbies), whereas other domains e.g., household tasks, does not provide examples. We categorised patients' responses in Table A into the most suitable MacDQoL domain e.g., for household tasks included cooking, gardening and use of powerpoints.

References for S7 File.

1. Mitchell J, Bradley C. Design of an individualised measure of the impact of macular disease on quality of life (the MacDQoL). Qual Life Res. 2004;13: 1163-1175.
