# Supplementary material for: Impacts of impaired face perception on social interactions and quality of life in age-related macular degeneration: A qualitative study and new community resources
Source: PLoS One. 2018 Dec 31;13(12):e0209218. doi: 10.1371/journal.pone.0209218 (PMC6312296; doi:10.1371/journal.pone.0209218)
Supplement: S8 File — Includes Supplementary Table A. (PDF) [file pone.0209218.s008.pdf]

Impacts of impaired face perception on social interactions and quality of life in age-related macular degeneration: A qualitative study and new community resources

Jo Lane[1], Emilie M. F. Rohan[2], Faran Sabeti[2,7], Rohan W. Essex[3], Ted Maddess[2], Amy Dawel[1], Rachel A. Robbins[4], Nick Barnes[5], Xuming He[6], Elinor McKone[1*]

PLoS ONE 20 December 2018

Interview 2 results for patient endorsement of content included in Faces and Social Life in AMD Information Sheet (Includes Table A).

Table A indicates the degree to which, in Interview 2, patients endorsed the inclusion of particular concepts in our *Faces and Social Life in AMD Information Sheet*. The table includes only statements for which the key information was eventually included in the Information Sheet. The final wording in the sheet may have been simplified or shortened as compared to statement listed in table.

Note Table A does not list all statements that patients were asked to consider. Some additional statements were excluded from the final information sheet because patient responses indicated the statements were poorly or confusingly worded (i.e., the patients couldn't understand them), or had too much information (and therefore it was not certain which part of the statement patients were endorsing). Further, some additional statements had included draft information about severity of AMD (along the lines, for example, that patients with less severe AMD would have no trouble seeing faces/expressions, or be fine seeing them close-up) which in fact turned out to be inaccurate once we had analysed the full interview

data from all participants. Another observation from patients' responses was that the information sheet should be kept purely to dealing with issues related to faces and social interactions: we found that any statements including any mention of other aspects of AMD (e.g., either medical details, or other aspects of vision loss such as driving) distracted patients by making them think the information sheet was about these topics as well, or AMD more broadly, and thus should include extensive other information about AMD (which is well-covered by existing AMD public information sheets). We also included three open ended questions about whether patients had other points they thought would be good to include, but these did not elicit any additional information.

In Table A, where total patient numbers do not equal 19 (the number who participated in Interview 2), this is because one or more patients: did not provide specific feedback about the statement (e.g., they described their own experience or were distracted by components of the question that were not relevant for present purposes, e.g., reading and driving); said they couldn’t comment because they did not have personal experience regarding the statement; or were not asked the question (due to fatigue, being distracted or they did not endorse a previous relevant statement).

Table A. Patient Endorsement of Including Particular Statements in the Information Sheet.

Statement read to patients	# patients endorsing
**Faces and social interactions**	
People living with AMD can have problems with faces. This includes recognising who other people are, and also with seeing their facial expressions.	18/18
There is a lot of variation across the visual abilities of AMD patients.... Some find bright light useful ... whereas others are sensitive to bright light. *[Full statement included additional material on other topics; patient transcripts have been used to extract specific endorsement of information about variability.]*	10/16
Some people with AMD may appear disengaged in social situations, this may be because they cannot see who is in a room.	15/15
People with AMD [1] may also be unable to see a person's facial expressions i.e., whether someone looks happy, sad or bored. Because they cannot see facial expressions, they might miss social cues. For example, someone might be looking bored but the person with AMD can't see this so they keep on talking, or a person might be just having a joke and is smiling when they say something, but the person with AMD takes it seriously.	15/16
Do you think it would be helpful to let others know that social situations can be tricky because of vision loss in AMD? *['Yes' responses]*	10/11
Where a person with AMD has trouble with faces this can lead to a variety of difficulties in social settings. For example, people may feel less confident in social situations and they may also feel frustrated or embarrassed that they cannot recognise people from their face. These feelings can impact how a person with AMD behaves in social situations which may include becoming less interactive and engaged and more passive. For example, they might wait for people to come to them rather than approaching other people in the room. It could also include waiting for other people to speak first, before they speak.	18/18
Vision loss in AMD can affect a person's confidence in social situations	18/19
**What can be done to help?**	
So, what can be done to help? First, there are some strategies a person with AMD might try out by themselves to help with recognising people. Strategies that might be helpful to try include: waiting until the other person speaks and recognising their voice; or trying to recognise the person from distinctive clothing, or their hair, or their body shape because these things don't require such fine vision as recognising their face. Be aware, however, that these strategies do not always work, e.g., the other person may not speak, or they might have changed their appearance in some way (like their hairstyle or lost weight), or they may wear similar clothing to lots of other people.	19/19
For recognising expressions, strategies that the person with AMD might find helpful to try include: listening to tone of voice, noticing body language and context e.g., is another person engaging with others or sitting alone? Be aware, however, that these strategies do not always work e.g., people may not be open about the way they feel and behave in a way that does not indicate their emotions, and vision loss in AMD will make it difficult to pick up subtle cues from the facial expressions that indicate a person's true emotions.	18/18
There are also strategies people around the person with AMD — such as family members, friends, and carers — can try when they are having trouble seeing faces. These strategies involve giving the person extra information to help them identify who people are, and what their expressions are showing; e.g., you could:<br>- Say "Hi Bob" to identify a person aloud as they approach, or provide other clues about the person such as "How did the trip to the coast with Mary go last week?"<br>- Whisper the name of a person approaching<br>- Identify out loud familiar people sitting around the room that are too far away for the person with AMD to recognise. For example, "Aunty Jo is sitting at the back of the room with her husband". This may encourage the person with AMD to move around a room and be more engaged in social situations if they know who is present.<br>- For facial expressions and mood, say "Uncle Bruce is looking down today" or "Bob is sitting on his own and he looks tired" or "Joan had a big smile on her face when she said that, I think she is having a joke with you".	19/19
**Others don't understand/ concerns about faking it**	
The fact that AMD is invisible to other people can be difficult and confusing. For example, others can see you doing some visual tasks (walking around) and they might forget you cannot do other visual tasks (read and see faces). Do you think explaining this to others might help? *['Yes' responses]*	19/19
Because of these inconsistencies across visual tasks, other people may think a person with AMD is faking their vision loss (or people with AMD might worry that others might think this). Do you think explaining this to others might help? *['Yes' responses]*	14/14

Notes: [1] Wording originally said "with moderate AMD" but patients endorsed the statement for all levels of vision loss.
